# Risk Factors for Adverse Prognosis and Death in American Visceral Leishmaniasis: A Meta-analysis

**DOI:** 10.1371/journal.pntd.0002982

**Published:** 2014-07-24

**Authors:** Vinícius Silva Belo, Claudio José Struchiner, David Soeiro Barbosa, Bruno Warlley Leandro Nascimento, Marco Aurélio Pereira Horta, Eduardo Sérgio da Silva, Guilherme Loureiro Werneck

**Affiliations:** 1 Departamento de Endemias Samuel Pessoa, Escola Nacional de Saúde Pública Sergio Arouca, Fundação Oswaldo Cruz, Rio de Janeiro, Rio de Janeiro, Brasil; 2 Departamento Básico—Área da Saúde—Campus Governador Valadares, Universidade Federal de Juiz de Fora, Governador Valadares, Minas Gerais, Brasil; 3 Departamento de Parasitologia, Universidade Federal de Minas Gerais, Belo Horizonte, Minas Gerais, Brasil; 4 Departamento de Epidemiologia e Métodos Quantitativos em Saúde, Escola Nacional de Saúde Pública Sergio Arouca, Fundação Oswaldo Cruz, Rio de Janeiro, Rio de Janiero, Brasil; 5 Campus Centro-Oeste Dona Lindu, Universidade Federal de São João del Rei, Divinópolis, Minas Gerais, Brasil; 6 Departamento de Epidemiologia, Instituto de Medicina Social, Universidade do Estado do Rio de Janeiro, Rio de Janeiro, Rio de Janeiro, Brasil; Institute of Tropical Medicine, Belgium

## Abstract

**Background:**

In the current context of high fatality rates associated with American visceral leishmaniasis (VL), the appropriate use of prognostic factors to identify patients at higher risk of unfavorable outcomes represents a potential tool for clinical practice. This systematic review brings together information reported in studies conducted in Latin America, on the potential predictors of adverse prognosis (continued evolution of the initial clinical conditions of the patient despite the implementation of treatment, independent of the occurrence of death) and death from VL. The limitations of the existing knowledge, the advances achieved and the approaches to be used in future research are presented.

**Methods/Principal Findings:**

The full texts of 14 studies conforming to the inclusion criteria were analyzed and their methodological quality examined by means of a tool developed in the light of current research tools. Information regarding prognostic variables was synthesized using meta-analysis. Variables were grouped according to the strength of evidence considering summary measures, patterns and heterogeneity of effect-sizes, and the results of multivariate analyses. The strongest predictors identified in this review were jaundice, thrombocytopenia, hemorrhage, HIV coinfection, diarrhea, age <5 and age >40–50 years, severe neutropenia, dyspnoea and bacterial infections. Edema and low hemoglobin concentration were also associated with unfavorable outcomes. The main limitation identified was the absence of validation procedures for the few prognostic models developed so far.

**Conclusions/Significance:**

Integration of the results from different investigations conducted over the last 10 years enabled the identification of consistent prognostic variables that could be useful in recognizing and handling VL patients at higher risk of unfavorable outcomes. The development of externally validated prognostic models must be prioritized in future investigations.

## Author Summary

In contrast to other clinical presentations of leishmaniasis in Latin America, American visceral leishmaniasis (VL) can lead to death in 5-10% of patients under treatment. The fatality rates associated with this disease have remained stable at a high level over the years in Brazil and are neither recorded in under-treatment patients from endemic countries of the Old World nor from non-endemic countries where such cases are imported. Since VL-induced lethality can occur even after the implementation of recommended therapy, the understanding of individual, clinical and laboratory factors that predispose to an unfavorable outcome might represent an important feature for informing better practice in the clinical management of cases. The present systematic review with meta-analysis brings together information on various prognostic variables associated with the severity of VL. Potential predictors identified in the studies surveyed were grouped according to the strength of evidence available, and 13 were considered to be of significant relevance. The gaps in the existing knowledge and the need for the development of externally validated prognostic models were also discussed. The results presented herein could be useful in identifying patients at higher risk of unfavorable evolution or death from VL, and might provide an aid in decision-making regarding the clinical management of VL cases.

## Introduction

Visceral leishmaniasis (VL) constitutes a serious public health problem in endemic regions, especially in the Indian sub-continent, in North and East Africa, and in South America. However, VL is one of the most neglected diseases in the world [1], closely associated with poverty, for which effective and affordable chemotherapies remain scarce [2], [3]. In Brazil, American VL was originally confined almost entirely to rural areas in the northeast of the country, but since the 1980s the disease has spread to large cities in the northeast, southeast and center-west regions of the country [4]. During the first decade of the 21st century, some 40,000 cases of VL and 2,500 VL-related deaths were reported in the country with no signs of a significant reduction in the fatality rates [5], [6].

In the Americas, the transmission of VL to humans occurs through the bite of female phlebotomine sandflies of the genus *Lutzomyia*, which hosts the promastigote form of *Leishmania infantum* [7]. After a relatively long incubation period of 3 to 8 months, the disease manifests itself through signs and symptoms that include irregular or remittent fever, cough, tiredness, weakness, loss of appetite and weight, together with those caused by invasion of the parasite into the phagocytic system such as enlargement of lymph nodes, liver and spleen [8]. The evolution of VL varies from case to case, and some infected individuals may never exhibit any signs of the disease [9], [10]. In cases of VL-related mortality, the outcome results predominantly from hemorrhage or co-infection [11].

Treatment options for VL in Brazil are pentavalent antimonial compounds and formulations of amphotericin B [12]. Although amphotericin B exhibits stronger antileishmanial activity than pentavalent antimonials, the treatment practice employed in Latin America is based on weak scientific evidence [4] and may induce parasite resistance [13] or be subject to host-related limitations associated with unresponsiveness, drug toxicity or prolonged parenteral administration [14].

The lack of reduction in the fatality rates of VL in Brazil can be explained not only by the limitations in therapy applied and the delay in diagnosis [12], but also by the lack of adequate management provided to individuals at higher risk of an unfavorable evolution of the disease. In this context, the identification of prognostic factors associated with VL might be a valuable tool for clinical practice. Prognostic factors are defined as variables that predict the course of a disease, possible outcomes and the frequency with which they can be expected to occur. Knowledge about such factors is essential in medicine, prompting the selection of the most appropriate diagnostic tests and treatments to be applied, assisting in the development of new medical interventions, aiding in the monitoring of disease progression, and facilitating the counseling of patients regarding their future health condition [15]–[17]. In the case of VL, prognostic indicators of disease severity could also be used to establish if treatment should be carried out in primary health care units or in specialized care centers, and would be of considerable value in prescribing specific interventions for patients at most risk of a lethal outcome [12], [18].

Generally, prognostic factors have received less research attention than etiological factors and therapeutics [15], [19], although in some medical fields, particularly those related to oncology, several prognostic models have been published [20], [21]. In Brazil, a number of studies have been performed with the purpose of identifying individual, clinical and laboratory factors associated with poor evolution of VL and/or lethal outcome [12]. However, to the best of our knowledge, no systematic review articles have been published summarizing the current state of understanding of VL prognostic factors and indicating the most consistent predictors.

Considering the relevance of predictors of clinical evolution in reducing the number of VL-induced deaths, and the need for reliable prognostic models (developed and validated according to appropriate methodologies), the present systematic review with meta-analysis seeks to bring together information reported in studies of the potential predictors of death and other adverse outcomes of American VL. In addition, based on the analysis of the limitations of the published studies and of existing knowledge we propose possible improvements that might be incorporated into future research.

## Methods

### Search for publications, inclusion/exclusion criteria and data extraction

**Figure 1:**
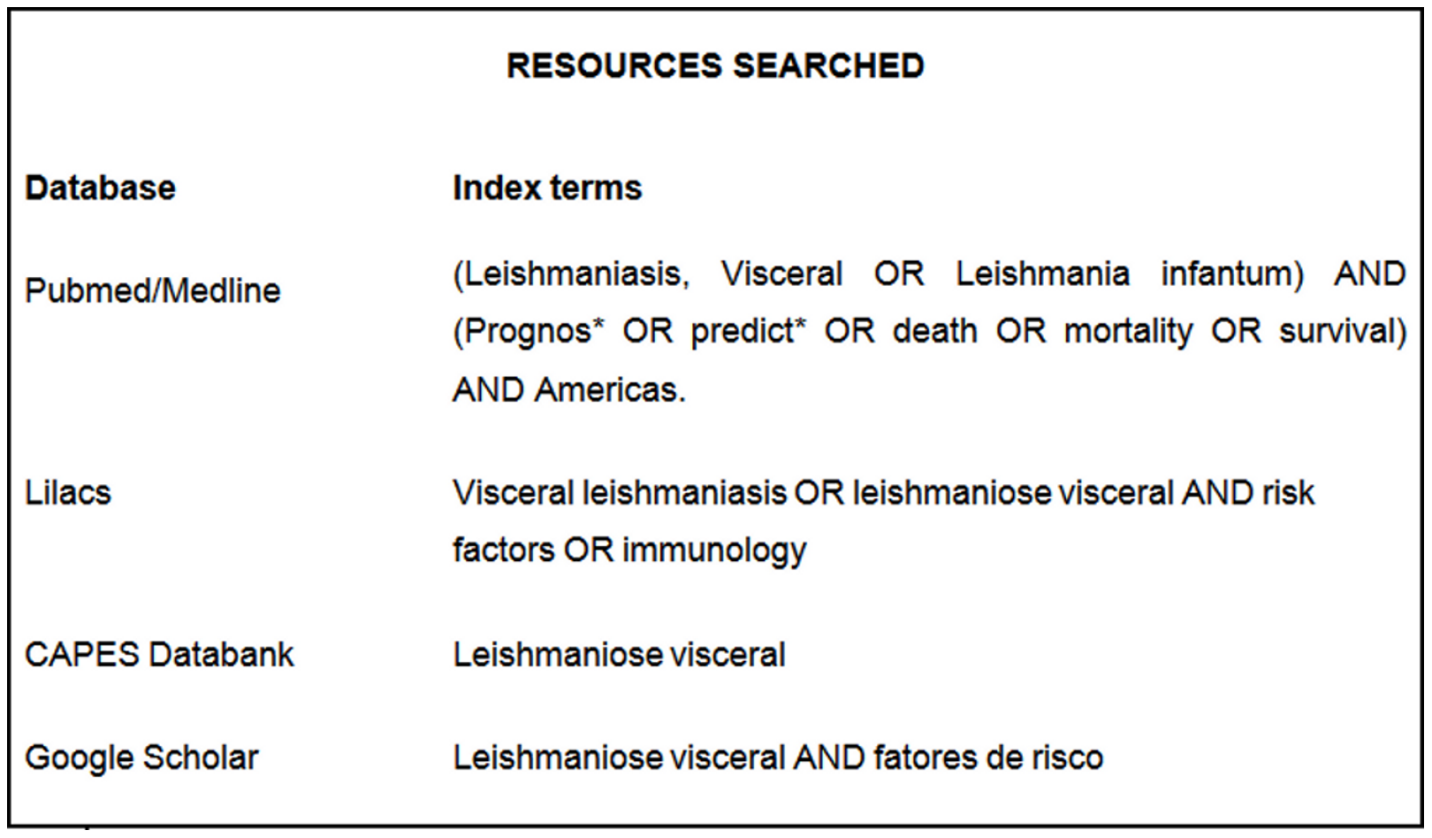
Index terms used in the search of published data. CAPES databank is a collection of theses and dissertations published by Brazilian academic institutions and assembled by Coordenação de Aperfeiçoamento de Pessoal de Nível Superior.

Independent literature searches were conducted between March and September 2011 by two of the authors (VSB and DSB) using the databanks and keywords listed in Fig. 1. Additional studies were identified by contacting experts in the field and by searching reference lists within selected publications. The titles and abstracts of all articles identified in the searches were subjected to an initial evaluation, and the full texts of those considered potentially relevant by at least one of the authors were analyzed.

The systematic review encompassed epidemiological studies containing data that allowed us to estimate measures of association relating to predictors of death or of adverse prognosis independent of the occurrence of death (sets of signs and/or symptoms characterizing the continued evolution of the initial clinical conditions of the patient following the implementation of treatment) in individuals diagnosed with VL. No restrictions were made regarding the age or gender of the patients or of the language of the publication. The exclusion criteria proposed were (i) studies performed outside Latin America; (ii) reports published as proceedings of symposiums or conferences; (iii) studies restricted to the description of signs and symptoms observed in VL-infected individuals without comparisons regarding the evolution of the disease; (iv) studies that simply described the existence of statistically significant (or not) associations without reporting at least the calculated *P* values or crude data that made possible the calculation of effect sizes (provided such information had not been obtained directly from the authors); (v) studies containing confusing text or incomprehensible analyses; (vi) studies exhibiting bias or inconsistencies that invalidated the results; and (vii) studies of prognostic factors related to genetic features or to quantification of cytokines.

The extraction of data from the publications was performed by one of the authors (VSB) and verified by the co-authors. Attempts were made to contact the authors of original reports when further information was required in order to calculate measures of association for possible inclusion in the meta-analysis. Data pertaining to individual patients were not requested.

### Information gathering and synthesis

The selected studies were separated into two main groups according to the outcomes, namely: (i) adverse evolution of the disease independent of death (as defined in the last section), (ii) evolution of the disease resulting in death. The first group of studies encompassed various possible outcomes and the information concerning each of the clinical or laboratory predictors identified was, if considered plausible (i.e. if the issue examined, the cut-off points and methods of analysis were not divergent), combined through meta-analysis of one sized *P*-values using the Stouffer method, weighted proportionally to the inverse of the study squared standard error [22]. In the second group, meta-analysis of measures of association were performed when cut-off points for defining variable categories employed in primary studies had close values. In this case, the effect-sizes adjusted by the greatest number of variables in each study were pooled regarding the odds ratio (OR). However, when there was divergence regarding the cut-off points, or when the predictors were defined differently but were relatively similar, meta-analysis of *P*-values was carried out as for the first group. For both groups of studies, we conducted theoretical discussions about variables that could not be submitted to meta-analysis, either because of the small number of studies involved or because of the non-uniform manner in which the data were presented or analyzed among the primary studies.

Measures of association were combined using the random effects model, except when the number of studies was less than four in which case the fixed effects model was employed [3]. The occurrence of heterogeneity in measures of effect between studies was analyzed using the *I^2^* test, which describes the percentage of total variation across studies associated with real dispersion in effect-sizes (inter-study variation) rather than random error (intra-study variation). For each prognostic factor, the studies were separated according to the ages of the participants (adults and children) and evaluations were performed separately for each group. When the measures of association were similar in the two groups the data were combined, otherwise the combination of data was performed only within the specific group.

Meta-P software was employed for the meta-analysis of *P*-values, while CMA software version 2.0.057 was used for all other meta-analyses.

### Criteria for defining the strength of predictors

The relative strength of each of the clinical and laboratory variables as a predictor of the severity of VL was evaluated according to defined criteria which were, in decreasing order of weight: (i) force of summary measure obtained through meta-analysis; (ii) pattern of data (direction of association and heterogeneity in studies where the outcome was death); (iii) number of statistically significant studies in which the control for confounding variables had been performed; and (iv) pattern of associations in studies where the outcome was unfavorable clinical evolution independent of death.

### Limitations and susceptibility to bias of the included studies

**Figure 2:**
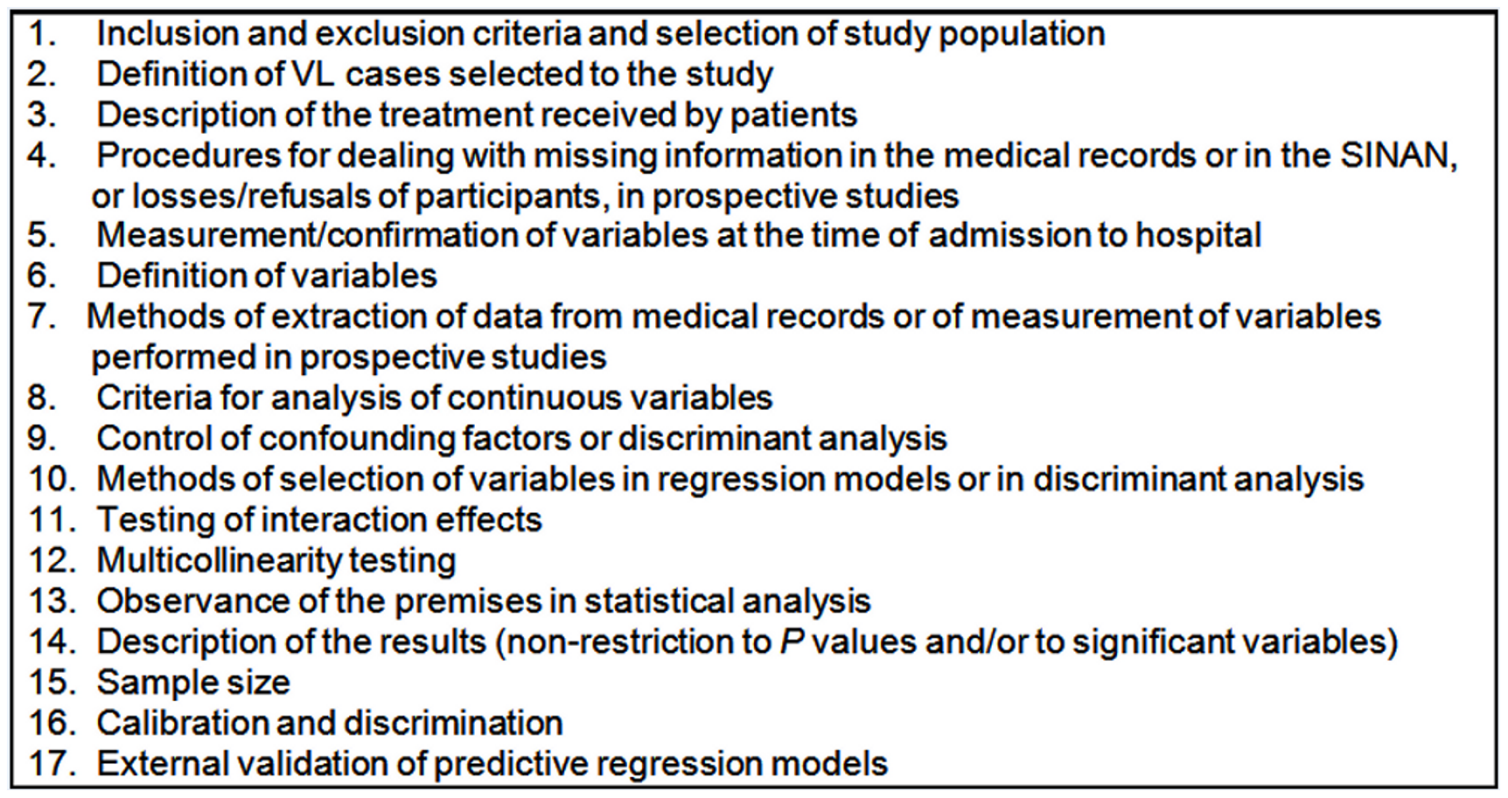
Conditions for assessment of the quality of the selected papers determined according to the principles described in the literature [15], [16], [23]–[26].

There is no universally accepted or standardized tool for the identification of limitations or potential risks of bias in the analysis and/or presentation of data in studies relating to prognostic factors. Thus, in order to analyze the quality of studies reviewed we opted to use five publications [15], [16], [23]–[25] describing principles and methods for the development of prognostic models. Additionally, the STROBE statement, the aim of which is to strengthen the reporting of observational studies in epidemiology, was used to complement these resources [26]. Based on these publications, a set of 17 conditions was established in order to evaluate the adequacy of the methodology employed and the clarity of presentation of the results described in the included studies (Fig. 2).

## Results

### Characteristics of the articles selected

**Figure 3:**
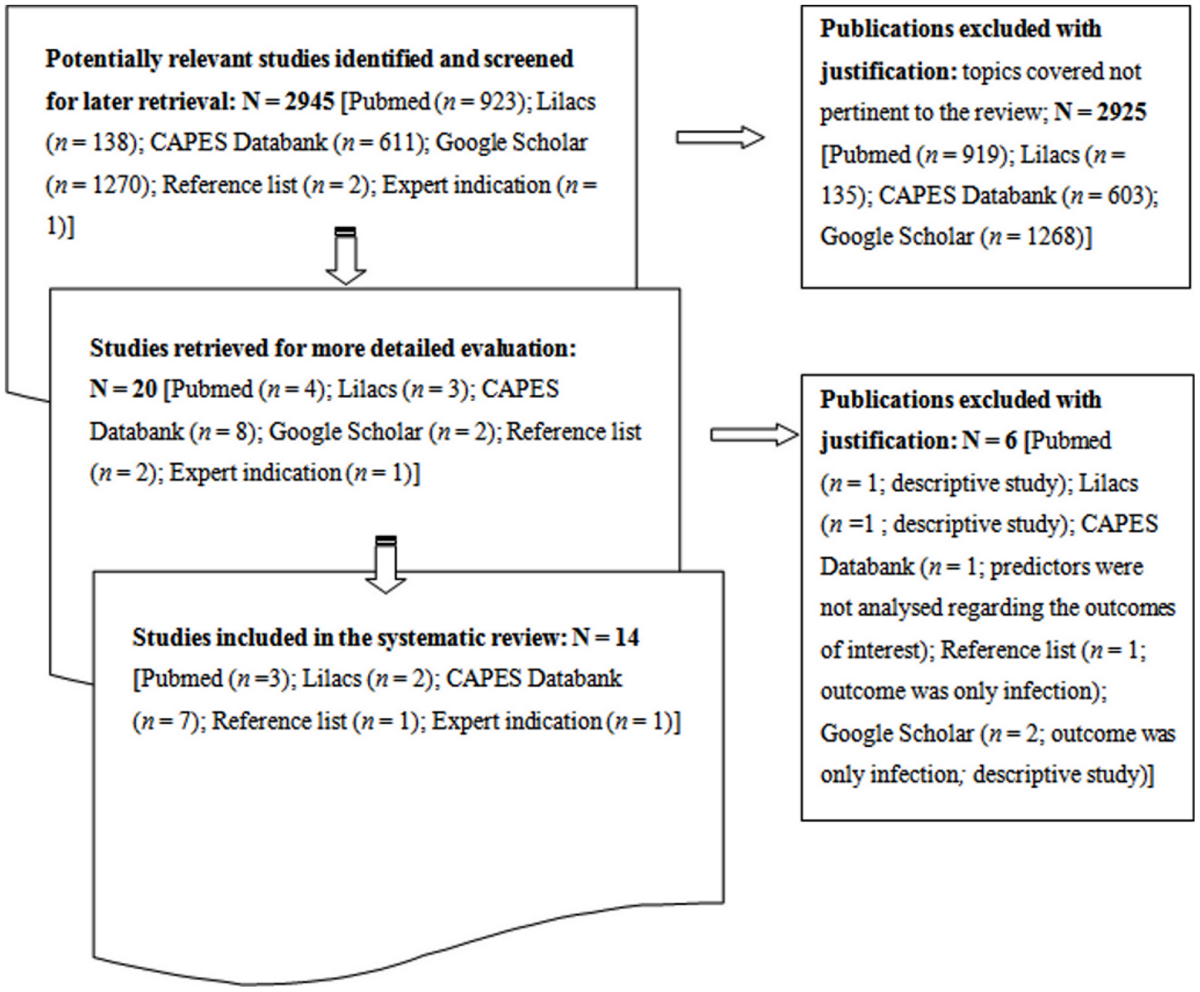
Flowchart representing the methodology employed in the selection of studies.

Of the 2945 studies identified and screened as part of the comprehensive survey, only 14 prognostic studies [11], [18], [27]–[38] complied fully with the inclusion criteria (Fig. 3). Although the survey covered studies conducted in all Latin America, the 14 selected publications originated from Brazil. Ten publications described death as the outcome of interest, while three referred to the clinical evolution of the patients independent of death, and one targeted both outcomes. The sources of information used in these studies were medical records (11/14), direct interviews with the patients during hospitalization (2/14) and the Brazilian Information System on Disease Notification (*Sistema de Informação de Agravos de Notificação*; SINAN; 1/14) as shown in Table S1.

### Strengths and weaknesses of the studies selected

**Figure 4:**
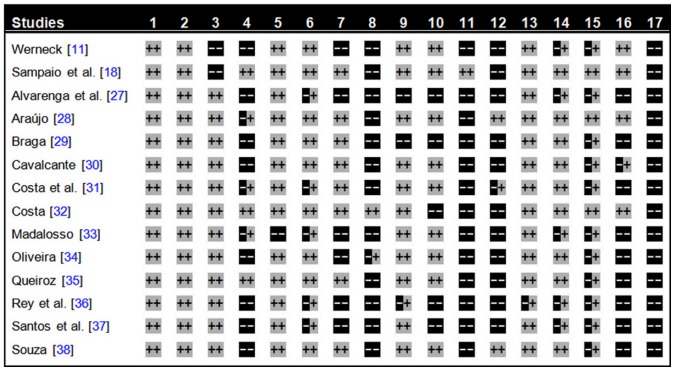
Assessment of the adequacy of the methodology employed and the clarity of presentation of the results described in the selected studies determined according to the conditions presented in the Figure 2 Legend: article described and adequately complied with the condition (two positive symbols), article did not refer to the procedure condition (two negative symbols), and article referred to the procedure but did not fully comply with the condition (a negative and a positive symbol).

Each of the 14 studies reviewed employed appropriate criteria for selecting the study populations and defining the cases, and all except one [36] observed fully the premises for the statistical analysis of the data. Only two studies [27], [29] failed to employ any control for confounding factors and to describe the treatment adopted (which was always based on the recommendations of Brazilian Ministry of Health [12]), although a number of studies presented limitations regarding the definitions of variables [27], [31], [33], [36], [37], extraction of data from medical records [11], [27], [33], [34], [36], [37], selection of variables for the regression models [27], [29], [32], [36], [37], and description of the results [11], [27], [33], [36], [37]. Eight articles failed to provide information regarding missing data in the medical records/SINAN or sample losses [11], [27], [29], [30], [34], [36]–[38] and three [18], [32], [35] of the six studies that described these aspects did not treat the matter in the correct manner. Only one study [32] employed adequate criteria for the stratification of continuous variables. The statistical power was generally low and the treatment of data and the description of the methods employed for the construction of models were poorly described in most articles. For example, testing of interaction effects was described in only one study [18], while multicollinearity testing was fully described in just two studies [28], [38]. Additionally, more than half of the studies (9/14) ignored completely calibration and discrimination procedures [27], [29], [31], [33]–[38]. None of the studies addressed the issue of validation of the predictive regression models in populations other than that of the original study (Fig. 4).

### Strength of predictors of adverse prognosis and death

All predictors of adverse evolution of VL and/or related mortality for which it was possible to perform meta-analysis (Text S1) are presented and classified according to strength in Table S2. Nine potentially strong predictors (Groups I and II in Table S2) were identified, namely, jaundice, thrombocytopenia, hemorrhage, HIV coinfection, diarrhea, severe neutropenia, age <5 years, age >40–50 years, dyspnoea and bacterial infection. All but the last three factors mentioned above presented summary measures significantly associated with mortality, consistency of effects in the direction of adverse evolution and mortality, and statistical significance in the majority of the multivariate analyses. While age >40–50 years, dyspnoea and bacterial infection were also strong predictors of death, their strength with respect to adverse evolution could not be assessed owing to the lack of studies exploring this outcome independent of death.

Apart from hepatomegaly, splenomegaly and weight loss, which were considered weak predictors, there was a predominance of statistically significant summary measures that showed, however, no significance in the majority of multivariate analyses (Groups III–V in Table S2).

Additionally, separate analyses of the variables in adults and children showed that there were no differences between the two groups except for the gender of participants and the interval between onset of symptoms and hospital admission, indicating that, in general, the predictors pointed in the same manner and direction independent of age group.

Prognostic factors that could not be submitted to meta-analysis did not form part of the classification of evidence adopted in this review. For example, Costa [32] reported that inappetence, kidney failure and high levels of alkaline phosphatase were highly associated with the risk of death, while other studies [29], [31] showed that VL-infected individuals with proteinuria had increased risk of unfavorable evolution and death. Moreover, Madalosso [33] demonstrated an association between mortality and positive myelogram, tuberculosis, dehydration, cardiovascular anomalies, asthenia, diabetes, splenectomy, myocardiopathy and abdominal pain. In addition, Costa et al. [31] demonstrated that VL-infected individuals with creatinine levels above 1.2 mg/100 mL exhibited high mortality risk, while Alvarenga et al. [27] showed that VL-infected individuals with comorbidities (HIV infection, liver and kidney diseases, cardiopathy, and other non-defined problems) had less chance of survival, similar to the findings of Araujo [28] for patients with other comorbidities (weakness and tuberculosis). Finally, Cavalcante [30] reported that individuals who recovered from VL within 20 days of treatment presented a higher mean eosinophil count as compared with those that did not recover, while individuals whose outcome was death exhibited higher mean values of prothrombin time and erythrocyte sedimentation rate compared with those that recovered. On the other hand, Braga [29] and Souza [38] showed that there was no significant difference between individuals that recovered and those that did not recover within 20 days of treatment regarding the mean lymphocyte count as well as when some cut-off point was used for this parameter.

## Discussion

The present systematic review identified, combined and analyzed information reported in studies addressing the factors associated with adverse prognosis of American VL and associated mortality. It was possible to identify a set of variables that ought to be considered in the clinical practice in order to improve disease management of patients and deserve further evaluation in future etiological and interventional studies in order to increase the empirical evidence on which to base their causal role.

The occurrence of jaundice was the strongest risk factor for severity of VL, demonstrating the relevance of hepatic impairment in disease progression. The association between jaundice and blood clotting disorders suggests the existence of a common hepatic mechanism [32], while liver dysfunctions in association with thrombocytopenia may lead to severe hemorrhage that could be responsible for the increased risk of death [39]. Considering that pentavalent antimonials, which represent the first-line of treatment of VL, are known to cause hepatotoxic side effects [40], VL-diagnosed individuals with jaundice or altered liver disease markers should be treated with amphotericin B-based pharmaceuticals rather than with antimonials. Inexplicably, this approach is not always followed, as exemplified by the patients investigated by Alvarenga et al. [27].

Hemorrhage was also a strong prognostic factor for adverse evolution of VL, and complications arising from this condition were major causes of death. Thus, the detection of bleeding at the first diagnosis or during the course of treatment is crucial in the identification of severity. According to Costa [32], hemorrhage is a consequence of the VL-induced inflammatory process, since pathogenesis of the disease is based on a cascade of events comprising activation of the inflammatory response, development of systemic endothelial lesions, activation of intravascular clotting, hypoperfusion, hypoxemia and, ultimately, cell death. Although the present review did not take into account the number of bleeding sites, it has been demonstrated that the greater the number of hemorrhagic points the higher is the risk of death [11], [32], suggesting that such relationship must be further investigated.

Thrombocytopenia was the second most important predictor of VL-induced death, although it is not possible to state with certainty if this variable is a cause or a consequence of hemorrhage. Splenic sequestration of platelets is possibly the main cause of a low platelet count [41], but this hypothesis only partially explains the disruption of homeostasis [32], and it has been suggested that thrombocytopenia is directly associated with the systemic inflammation induced by disseminated intravascular clotting [42]. From the reviewed data, it would appear that counts lower than 100,000 platelets/mm^3^ are indicative of high risk of adverse evolution, although a cut-off point of 50,000 platelets/mm^3^ is associated with an even higher risk. Thus, rather than attempting to define a standard limit of thrombocytopenia, it is of greater importance to assess each case separately in order to decide which is the most appropriate hemotherapeutic approach. In this context, the manual issued by the Ministry of Health of Brazil [12] recommends platelet transfusion only for VL patients presenting counts lower than 10,000 platelets/mm^3^.

*Leishmania*-HIV coinfection was a relevant prognostic factor for the adverse evolution of VL, since all studies analyzed and all multivariate analyses performed showed that coinfected individuals had a higher risk of poor prognosis. Considering that HIV induces the replication of *Leishmania*, that Th1-type immune response changes into Th2-type in both VL and HIV infection, and that HIV as well as *Leishmania* infect and multiply within cells of myeloid or lymphoid origin, the damaging effects of HIV and VL on the cellular immune system are not only synergistic but also reciprocally modulate pathogenesis [43]–[46]. According to Jarvis and Lockwood [47], the use of pentavalent antimonials is no longer recommended for HIV/VL-coinfected individuals owing to the high rates of failure and the level of toxicity associated with the treatment. These researchers emphasized the need for clinical tests to accelerate the development of more effective combined therapies and the planning of secondary prophylactic strategies. The Ministry of Health of Brazil [12] recommends HIV testing and the treatment with liposomal amphotericin B for all VL patients.

Together with hemorrhagic complications, the presence of bacterial infections is known to be an important cause of death among VL-infected individuals [11]. Even though this review included only studies that analyzed the occurrence of infections at the time of admission, the presence of coinfections represented a strong predictor of adverse evolution. This finding indicates the importance of preventing general infections and of treating VL patients isolated from individuals with bacterial infections, furthermore it calls attention to the damaging impact of late diagnosis on increased lethality of VL. Unfortunately, a large proportion of patients seeking medical assistance at hospitals or health units already presented opportunist infections and, possibly, VL at an advanced stage.

Severe neutropenia, characterized by the cut-off point of 500 neutrophils/mm^3^ [48], also constituted a strong predictor of VL severity. Patients with this condition had a higher risk of VL-related death, possibly because they were more susceptible to bacterial infections. In such cases, the use of antibiotics and the constant monitoring of this parameter are mandatory throughout the course of treatment.

Interestingly, diarrhea was a strong predictor of mortality. However, according to Werneck et al. [11], the occurrence of melena may be incorrectly interpreted as diarrhea or enteric microorganisms may be responsible for the sepsis associated with clotting abnormalities. Dyspnoea was also a good indicator of increased risk of unfavorable evolution of VL, and assessment of this condition, together with that of diarrhea, should be a routine priority in clinical practice since evaluation of these two parameters is rapid and straightforward, and their presence is possibly the result of more severe complications [35].

Although age of the subject was a strong indicator of poor clinical course of VL, most studies included in the review used dissimilar cut-off points, and few analyzed age as a continuous variable. Nevertheless, the data revealed that children of less than five years (especially those less than one year) and adults above 40 years (especially those older than 50 years) are more likely to have an adverse evolution. The distribution of lethality with peaks among children and older adults suggests that different factors may be involved in the acquisition of infections and complications at different ages [32]. In particular, the elderly are more frequently affected by comorbidities, such as cardiovascular diseases and weaker immunological resistance, which may increase the risk of death [49], [50]. On the other hand, children exhibit increased interleukin-10 levels and L-arginine secretion, which are factors associated with parasite persistence and greater VL severity. These parameters, coupled with the immaturity of the immune system, could explain the poor prognosis for this age group [42], [51]–[54].

Together with the strong prognostic factors of groups I and II (Table S2), it is worth considering the importance of the other groups of variables in the clinical evaluation of patients and for the purposes of improved disease management. For example, group III variables (Table S2) were statistically significant according to meta-analysis and some (but not the majority) of the multivariate analysis. In particular, the presence of edema emerged as a relevant indicator of VL severity since, although not significant in half of the multivariate analyses, it was strongly associated with death, similarly to the presence of vomiting.

The reduced strength of some relationships may be attributed to the specific therapeutic measures employed in some cases. For instance, individuals presenting hemoglobin levels below 7 g/dL would have received transfusions of packed red cells, as recommended by the Ministry of Health of Brazil [12], and this strategy may have diminished not only the degree of anemia but also the strength of the association between hemoglobin and VL severity. Nevertheless, low hemoglobin concentration was strongly associated with death and, therefore, it represented a relevant prognosis factor. Regarding undernutrition, there is evidence suggesting that this condition is more a consequence of the wasting syndrome in VL rather than a risk factor for severity. Furthermore, the control of *Leishmania* replication by the adaptive immunosystem, particularly by Th1 cells, of undernourished patients could explain the lack of association between undernutrition and mortality risk [32]. Some other laboratory variables, such as leukocyte count and levels of albumin, alanine transaminase (ALT) and aspartate transaminase (AST), constituted prognosis factors of intermediary evidence in the prediction of poor prognosis.

Several potentially relevant variables could not be included in the categories of evidence proposed herein because of the scarcity of studies. Among these are factors that can be readily assessed in clinical practice with minimal cost and must be better evaluated in future research, for example, mean cell volume, eosinophil count, serum creatinine, inappetence, weakness or asthenia, dehydration, lymphadenopathy and occurrence of comorbidities such as diabetes, tuberculosis, heart or renal diseases and dengue fever. In this context, it is noteworthy that the influence of helminthiasis on the clinical evolution of VL was not investigated in any of the reviewed studies even though infection by intestinal parasitic worms is highly prevalent in urban and rural areas of Brazil [55], [56]. It is well known that helminths can modulate and even suppress the immune response and, consequently, modify the clinical manifestations of diseases associated with the immune system [57], [58], hence this topic also should be included in future research. Other variables that require more specific and expensive tests, including myelogram, cardiovascular abnormalities, bilirubin levels, prothrombin time and partial thromboplastin time, have also received little research attention.

The present review provides a reliable source of information for the identification of risk factors of adverse prognosis and mortality in VL and should be used as an aid in decision-making in clinical practice. It is important to emphasize, however, that the results presented herein do not directly allow the creation and validation of prognostic scores based on the signs and symptoms presented by patients. Thus, studies should be carried out with the specific purpose of developing such scores and performing external validation of prognostic models already proposed, along with the incorporation of prognostic factors or additional biomarkers as recommended by Pencina et al. [59]. Assessment of the quality of the studies reviewed herein revealed that only five developed scores based on data obtained from the study populations, and no external validation of any kind was performed in these investigations. Prognostic models may present poor reproducibility and predictive performance when applied to other populations owing to the possibility of overfitting, the exclusion of some significant predictor, or differences between the characteristics of patients, health services or diagnostic methods [23]. According to Steyerberg et al. [60], a prognostic model is only useful if it is able to predict with accuracy the outcome of a patient who was not a member of the source population, i.e. the cohort employed in the development of the model, and studies that do not include at least some form of internal validation procedure (such as cross validation or bootstrapping) are rarely acceptable for publication. The manual issued by the Ministry of Health of Brazil [12] describes the implementation of a validation of the prognostic model developed by Costa [32], but does not include details regarding the procedures employed. For this reason, it is not possible to evaluate the score structure proposed in the model or to evaluate its potential applicability. However, this constitutes the first step in the formulation of a consistent prognostic model, with an impact that could be properly assessed, for application in different contexts in Brazil.

Concerning other limitations in the analyzed studies, the procedures adopted to deal with the problem of missing information from medical records were generally unclear. According to Little and Rubin [61], restricting an analysis to participants presenting complete records not only reduces the statistical power of the study but may also introduce bias. The pitfalls caused by missing data can be circumvented by the use of sophisticated statistical approaches especially designed for the imputation of missing information [62]–[65]. Such procedures should be employed as an alternative in all future studies whenever a set of values of variables are absent from a cohort.

The majority of studies considered in the present review failed to define the criteria adopted for the stratification of continuous variables. The quality of studies could be improved by adoption of credible and unequivocal clinical and analytical stratification criteria [66], or by analyzing continuous variables according to their original scale [67], [68] and by the implementation of appropriate procedures for the analysis of the functional form of their relationship with the outcome [69], [70].

Although the majority of the reviewed studies can be considered acceptable with respect to the adequacy of case definitions, statistical methods and multivariate analyses adopted, there were limitations in the models in cases where no interaction or multi-colinearity tests between the predictor variables were performed. In most of the studies, various prognostic factors were analyzed and many of them could be correlated, thereby producing the same explanation of variability in outcome [71]. In such cases, it is not correct to maintain all of the correlated variables in modeling procedures and, in view of the low statistical power of these studies, the exclusion of redundant explanatory variables would be helpful in increasing the accuracy of the multivariate model.

Considering the limitations of the present review, none of the studies conducted in other parts of the world were analyzed since those studies would reflect specific clinical, social and epidemiological characteristics distinct from those of VL in the Americas. Other relevant issues included the problem of combining data acquired from distinct populations (in terms of areas and characteristics) as well as the inability to explore the causes of heterogeneity of effect sizes between studies, and the impracticality of determining the existence of publication bias. Most studies described the results for all of the variables analyzed, but four articles did not provide data regarding some associations, particularly for non-significant variables, and this may have modified the true effect of some of the calculated summary measures. The force of these measures may also have been overestimated because of the use of odds ratio as a proxy for the relative risk [72], [73]. Additionally, there is the limitation regarding the sources of information, since most of the primary studies used retrospective data collected from medical records. The consistency and accuracy of such data is often a topic of discussion among researchers [74] because of the differences that exist in standards and in methods of registering data from one hospital to another. This does not mean that the use of medical records for research purposes should be abandoned, but that information derived from them should be examined with caution, and that those responsible for managing and for completing the records should be encouraged to improve the quality of information provided.

This is the first systematic review with meta-analysis on the prognosis factors relating to VL severity. The integration of information from different investigations conducted in Brazil in the last 10 years led to the identification of consistent predictor variables that might be useful in clinical practice for designing distinct therapies for patients at risk of an unfavorable outcome of the disease. The analysis of the quality of the published studies may be of assistance in future research, since positive features have been highlighted while logical criticism of the flaws, mainly relating to the external validation of multivariate prognostic models, has been offered. Similar assessments in different regions of the globe would be highly relevant since lethality of VL and the impact of this disease on our society can only be diminished by using consistent evidence-based medical approaches.

## Supporting Information

Table S1 Main characteristics of the studies included in the systematic review on prognostic factors relating to visceral leishmaniasis (VL) severity.
(DOC)

Table S2 Predictors, classified according to strength, of unfavorable clinical evolution independent of death and mortality for American visceral leishmaniasis identified in this systematic review.
(DOC)

Text S1 Forest plots for the variables submitted to meta-analysis.
(DOCX)

Text S2 PRISMA checklist [75].
(DOC)

## References

[1] Yamey G, Torreele E (2002) The world's most neglected diseases. BMJ 325: 176–177. 12142292 10.1136/bmj.325.7357.176 PMC1123710

[2] Bern C, Maguire JH, Alvar J (2008) Complexities of Assessing the Disease Burden Attributable to Leishmaniasis. PLoS Negl Trop Dis 2: e 313. 10.1371/journal.pntd.0000313 PMC2569207 18958165

[3] Belo VS, Werneck GL, Barbosa DS, Simões TC, Nascimento BW, et al (2013) Factors associated with visceral leishmaniasis in the americas: a systematic review and meta-analysis. PLoS Negl Trop Dis 25: e2182. 10.1371/journal.pntd.0002182 PMC3636096 23638203

[4] Romero GA, Boelaert M (2010) Control of visceral leishmaniasis in Latin America-A systematic review. PLoS Negl Trop Dis 4: e 584. 10.1371/journal.pntd.0000584 PMC2808217 20098726

[5] Harhay MO, Olliaro PL, Costa DL, Costa CH (2011) Urban parasitology: visceral leishmaniasis in Brazil. Trends Parasitol 27: 403–409. 21596622 10.1016/j.pt.2011.04.001

[6] Ministry of Health of Brazil (2012) Sistema de Informação de Agravos de Notificação. Ministério da Saúde, Secretaria de Vigilância em Saúde, Brasília. Available from: http://dtr2004.saude.gov.br/sinanweb/. Accessed 17 Dec 2012.

[7] Reithinger R, Brooker S, Kolaczinski JH (2009) Visceral leishmaniasis: time to better use existing resources. Lancet 374: 1330. 19837253 10.1016/S0140-6736(09)61825-0

[8] Chappuis F, Sundar S, Hailu A, Ghalib H, Rijal S, et al (2007) Visceral leishmaniasis: what are the needs for diagnosis, treatment and control? Nat Rev Microbiol 5: 873–882. 17938629 10.1038/nrmicro1748

[9] Evans TG, Teixeira MJ, McAuliffe IT, Vasconcelos I, Vasconcelos AW, et al (1992) Epidemiology of visceral leishmaniasis in northeast Brazil. J Infect Dis 166: 1124–1132. 1402024 10.1093/infdis/166.5.1124

[10] Silveira FT, Lainson R, De Souza AA, Campos MB, Carneiro LA, et al (2010) Further evidences on a new diagnostic approach for monitoring human *Leishmania (L.) infantum chagasi* infection in Amazonian Brazil. Parasitol Res 106: 377–386. 19946708 10.1007/s00436-009-1672-x

[11] Werneck GL, Batista MS, Gomes JR, Costa DL, Costa CH (2003) Prognostic factors for death from visceral leishmaniasis in Teresina, Brazil. Infection 31: 174–177. 12789476 10.1007/s15010-003-3139-9

[12] Ministry of Health of Brazil (2011) Leishmaniose visceral : recomendações clínicas para redução da letalidade. Ministério da Saúde, Secretaria de Vigilância em Saúde, Brasília, 120 p.

[13] Singh N, Kumar M, Singh RK (2012) Leishmaniasis: current status of available drugs and new potential drug targets. Asian Pac J Trop Med 5: 485–497. 22575984 10.1016/S1995-7645(12)60084-4

[14] Mondal S, Bhattacharya P, Ali N (2010) Current diagnosis and treatment of visceral leishmaniasis. Expert Rev Anti Infect Ther 8: 919–944. 20695748 10.1586/eri.10.78

[15] Moons KG, Royston P, Vergouwe Y, Grobbee D, Altman D (2009) Prognosis and prognostic research: what, why and how?. BMJ 338: b375. 19237405 10.1136/bmj.b375

[16] Mallett S, Royston P, Waters R, Dutton S, Altman DG (2010) Reporting performance of prognostic models in cancer: a review. BMC Med 8: 21. 20353579 10.1186/1741-7015-8-21 PMC2857810

[17] Riley RD, Hayden JA, Steyerberg EW, Moons KG, Abrams K, et al (2013) Prognosis Research Strategy (PROGRESS) 2: prognostic factor research. PLoS Med 10: e1001380. 23393429 10.1371/journal.pmed.1001380 PMC3564757

[18] Sampaio MJ, Cavalcanti NV, Alves JG, Filho MJ, Correia JB (2010) Risk factors for death in children with visceral leishmaniasis. PLoS Negl Trop Dis 4: e877. 21072238 10.1371/journal.pntd.0000877 PMC2970542

[19] Hemingway H, Croft P, Perel P, Hayden JA, Abrams K, et al (2013) Prognosis research strategy (PROGRESS) 1: a framework for researching clinical outcomes. BMJ 346: e5595. 23386360 10.1136/bmj.e5595 PMC3565687

[20] Hemingway H (2006) Prognosis research: why is Dr. Lydgate still waiting?. J Clin Epidemiol 59: 1229–1238. 17098565 10.1016/j.jclinepi.2006.02.005

[21] Reilly BM, Evans AT (2006) Translating clinical research into clinical practice: impact of using prediction rules to make decisions. Ann Intern Med 144: 201–209. 16461965 10.7326/0003-4819-144-3-200602070-00009

[22] Whitlock MC (2005) Combining probability from independent tests: the weighted Z-method is superior to Fisher's approach. J Evol Biol 18: 1368–1373. 16135132 10.1111/j.1420-9101.2005.00917.x

[23] Altman DG, Vergouwe Y, Royston P, Moons KG (2009) Prognosis and prognostic research: validating a prognostic model. BMJ 338: b605. 19477892 10.1136/bmj.b605

[24] Moons KGb, Altman DG, Vergouwe Y, Royston P (2009) Prognosis and prognostic research: application and impact of prognostic models in clinical practice. BMJ 338: b606. 19502216 10.1136/bmj.b606

[25] Royston P, Moons KG, Altman DG, Vergouwe Y (2009) Prognosis and prognostic research: Developing a prognostic model. BMJ 338: b604. 19336487 10.1136/bmj.b604

[26] Vandenbroucke JP, von Elm E, Altman DG, Gøtzsche PC, Mulrow CD, et al (2007) Strengthening the Reporting of Observational Studies in Epidemiology (STROBE): explanation and elaboration. PLoS Med 4: e297. 17941715 10.1371/journal.pmed.0040297 PMC2020496

[27] Alvarenga DG, Escalda PMF, Costa ASV, Monreal MTFD (2010) Leishmaniose visceral: estudo retrospectivo de fatores associados à letalidade. Rev Soc Bras Med Trop 43: 194–197. 20464152 10.1590/s0037-86822010000200017

[28] Araújo VE (2011) Análise da distribuição espaço-temporal da leishmaniose visceral e perfil clínico-epidemiológico dos casos e óbitoa, Belo-Horizonte, Minas Gerais, 1994 a 2009; Leishmaniose Visceral em Belo Horizonte: perfil clínico-epidemiológico de casos e óbitos do período de 2002 a 2009. [Doctorate Thesis] Belo Horizonte, MG: UFMG. 190p.

[29] Braga ASC (2007) Fatores associados à evolução clínica da leishmaniose visceral em crianças hospitalizadas em centro de referência de Belo Horizonte, 2001 a 2005. [MSC Dissertation] Belo Horizonte, MG: UFMG. 98p.

[30] Cavalcante MHL (2007) Leishmaniose visceral americana: aspectos clínicos e laboratoriais preditivos de prognóstico. [MSC Dissertation] Fortaleza, CE: UECE, 104p.

[31] Costa CHN, Werneck GL, Costa DL, Holanda TA, Aguiar GB, et al (2010) Is severe visceral leishmaniasis a systemic inflammatory response syndrome? A case control study. Rev Soc Bras Med Trop 43: 386–392. 20802936 10.1590/s0037-86822010000400010

[32] Costa DL (2009) Fatores de prognóstico na leishmaniose visceral: alterações clínicas e laboratoriais associadas à resposta imune, aos distúrbios da coagulação e à morte. [Doctorate Thesis] Belo Horizonte, MG: UFMG. 214p.

[33] Madalosso G (2006) Casos Autóctones de Leishmaniose Visceral Americana e Fatores Associados à Letalidade, Estado de São Paulo, Brasil, 1999 a 2005. [MSC Dissertation] São Paulo, SP: USP. 75p.

[34] Oliveira CDL (2006) Leishmaniose visceral na região metropolitana de Belo Horizonte: um estudo caso-controle. [MSC Dissertation] Belo Horizonte, MG: UFMG. 83p.

[35] Queiroz MJL (2002) Fatores prognósticos associados ao óbito por calzar em crianças internadas no Instituto Materno Infantil De Pernambuco (IMIP). [MSC Dissertation]. Recife, PE: IMIP. 130p.

[36] Rey LC, Martins CV, Ribeiro HB, Lima AAM (2005) Leishmaniose visceral americana (calazar) em crianças hospitalizadas de área endêmica. J Pediatr 81: 73–78. 15742090

[37] Santos MA, Marques RC, Farias CA, Vasconcelos DM, Stewart JM, et al (2002) Predictors of an unsatisfactory response to pentavalent antimony in the treatment of American visceral leishmaniasis. Rev Soc Bras Med Trop 35: 629–633. 12612746 10.1590/s0037-86822002000600014

[38] Souza GF (2007) Comparação de aspectos clínicos e diagnósticos da leishmaniose visceral entre portadores e não portadores do vírus da imunodeficiência humana. [MSC Dissertation] Belo Horizonte, MG: UFMG. 124p.

[39] Seaman J, Mercer AJ, Sondorp HE, Herwaldt BL (1996) Epidemic visceral leishmaniasis in southern Sudan: treatment of severely debilitated patients under wartime conditions and with limited resources. Ann Intern Med 124: 664–672. 8607595 10.7326/0003-4819-124-7-199604010-00007

[40] Lima EB, Porto C, Motta JOC, Sampaio RNR (2007) Tratamento da Leishmaniose Tegumentar Americana. An Bras Dermatol 82: 111–124.

[41] Varma N, Naseem S (2010) Hematologic changes in visceral leishmaniasis/kalaazar. Indian J Hematol Blood Transfus 26: 78–82. 21886387 10.1007/s12288-010-0027-1 PMC3002089

[42] Costa DL, Rocha RL, Carvalho RM, Lima-Neto AS, Harhay MO, et al (2013) Serum cytokines associated with severity and complications of kala-azar. Pathog Glob Health 107: 78–87. 23683334 10.1179/2047773213Y.0000000078 PMC4001482

[43] Olivier M, Badaro R, Medrano FJ, Moreno J (2003) The pathogenesis of Leishmania/HIV co-infection: cellular and immunological mechanisms. Ann Trop Med Parasitol 97 Suppl 1: 179–198. 14678636 10.1179/000349803225002561

[44] Cruz I, Nieto J, Moreno J, Cañavate C, Desjeux P, et al (2006) Leishmania/HIV co-infections in the second decade. Indian J Med Res 123: 357–388. 16778317

[45] Alvar J, Aparicio P, Aseffa A, Den Boer M, Cañavate C, et al (2008) The relationship between leishmaniasis and AIDS: the second 10 years. Clin Microbiol Rev 21: 334–359. 18400800 10.1128/CMR.00061-07 PMC2292576

[46] Okwor I, Uzonna JE (2013) The immunology of Leishmania/HIV co-infection. Immunol Res 56: 163–171. 23504228 10.1007/s12026-013-8389-8

[47] Jarvis JN, Lockwood DN (2013) Clinical aspects of visceral leishmaniasis in HIV infection. Curr Opin Infect Dis 26: 1–9. 23221770 10.1097/QCO.0b013e32835c2198

[48] Kim SY, Solomon DH, Liu J, Chang CL, Daniel GW, et al (2011) Accuracy of identifying neutropenia diagnoses in outpatient claims data. Pharmacoepidemiol Drug Saf 20: 709–713. 21567653 10.1002/pds.2157 PMC3142869

[49] Mueller Y, Mbulamberi DB, Odermatt P, Hoffmann A, Loutan L, et al (2009) Risk factors for in-hospital mortality of visceral leishmaniasis patients in eastern Uganda. Trop Med Int Health 14: 910–917. 19552645 10.1111/j.1365-3156.2009.02305.x

[50] Araújo VE, Morais MH, Reis IA, Rabello A, Carneiro M (2012) Early clinical manifestations associated with death from visceral leishmaniasis. PLoS Negl Trop Dis 6: e1511. 22347514 10.1371/journal.pntd.0001511 PMC3274500

[51] Groux H, Cottrez F, Rouleau M, Mauze S, Antonenko S, et al (1999) A transgenic model to analyze the immunoregulatory role of IL-10 secreted by antigen-presenting cells. J Immunol 162: 1723–1729. 9973435

[52] Slatter MA, Gennery AR (2008) Clinical Immunology Review Series: An approach to the patient with recurrent infections in childhood. Clin Exp Immunol 152: 389–396. 18373701 10.1111/j.1365-2249.2008.03641.x PMC2453200

[53] Munder M (2009) Arginase: an emerging key player in the mammalian immune system. Br J Pharmacol 158: 638–651. 19764983 10.1111/j.1476-5381.2009.00291.x PMC2765586

[54] Müller I, Hailu A, Choi BS, Abebe T, Fuentes JM, et al (2008) Age-related alteration of arginase activity impacts on severity of leishmaniasis. PLoS Negl Trop Dis 2: e235. 18478052 10.1371/journal.pntd.0000235 PMC2359854

[55] Fleming FM, Brooker S, Geiger SM, Caldas IR, Correa-Oliveira R, et al (2006) Synergistic associations between hookworm and other helminth species in a rural community in Brazil. Trop Med Int Health 11: 56–64. 16398756 10.1111/j.1365-3156.2005.01541.x

[56] Belo VS, Oliveira RB, Fernandes PC, Nascimento BW, Fernandes FV, et al (2012) Factors associated with intestinal parasitosis in a population of children and adolescents. Rev paul pediatr 30: 195–201.

[57] Helmby H (2009) Helminths and our immune system: friend or foe?. Parasitol Int 58: 121–127. 19223020 10.1016/j.parint.2009.02.001

[58] Newlove T, Guimarães LH, Morgan DJ, Alcântara L, Glesby MJ, et al (2011) Antihelminthic therapy and antimony in cutaneous leishmaniasis: a randomized, double-blind, placebo-controlled trial in patients co-infected with helminths and *Leishmania braziliensis* . Am J Trop Med Hyg 84: 551–555. 21460008 10.4269/ajtmh.2011.10-0423 PMC3062447

[59] Pencina MJ, D'Agostino RB Sr, D'Agostino RB Jr, Vasan RS (2008) Evaluating the added predictive ability of a new marker: from area under the ROC curve to reclassification and beyond. Stat Med 27: 157–172. 17569110 10.1002/sim.2929

[60] Steyerberg EW, Moons KG, van der Windt DA, Hayden JA, Perel P, et al (2013) Prognosis Research Strategy (PROGRESS) 3: prognostic model research. PLoS Med 10: e1001381. 23393430 10.1371/journal.pmed.1001381 PMC3564751

[61] Little RJ, Rubin DB (2002) A taxonomy of missing-data methods In: Statistical Analysis with Missing Data. New York: Wiley, 19–23.

[62] Rubin DB (1987) Multiple Imputation for Nonresponse in Surveys. New York: John Wiley.

[63] Barnard J, Meng XL (1999) Applications of multiple imputation in medical studies: from AIDS to NHANES. Stat Methods Med Res 8: 17–36. 10347858 10.1177/096228029900800103

[64] Sterne JA, White IR, Carlin JB, Spratt M, Royston P, et al (2009) Multiple imputation for missing data in epidemiological and clinical research: potential and pitfalls. BMJ 338: b2393. 19564179 10.1136/bmj.b2393 PMC2714692

[65] Lee KJ, Carlin JB (2010) Multiple imputation for missing data: fully conditional specification versus multivariate normal imputation. Am J Epidemiol 171: 624–632. 20106935 10.1093/aje/kwp425

[66] Naggara O, Raymond J, Guilbert F, Roy D, Weill A, et al (2011) Analysis by categorizing or dichotomizing continuous variables is inadvisable: an example from the natural history of unruptured aneurysms. AJNR Am J Neuroradiol 32: 437–440. 21330400 10.3174/ajnr.A2425 PMC8013096

[67] Altman DG, Lausen B, Sauerbrei W, Schumacher M (1994) Dangers of using “optimal” cutpoints in the evaluation of prognostic factors. J Natl Cancer Inst 86: 829–835. 8182763 10.1093/jnci/86.11.829

[68] Royston P, Altman DG, Sauerbrei W (2006) Dichotomizing continuous predictors in multiple regression: a bad idea. Stat Med 25: 127–141. 16217841 10.1002/sim.2331

[69] Sauerbrei W, Royston P, Binder H (2007) Selection of important variables and determination of functional form for continuous predictors in multivariable model building. Stat Med 26: 5512–5528. 18058845 10.1002/sim.3148

[70] Barrio I, Arostegui I, Quintana JM (2013) Group IC (2013) Use of generalised additive models to categorise continuous variables in clinical prediction. BMC Med Res Methodol 13: 83. 23802742 10.1186/1471-2288-13-83 PMC3716996

[71] Tu YK, Kellett M, Clerehugh V, Gilthorpe MS (2005) Problems of correlations between explanatory variables in multiple regression analyses in the dental literature. Br Dent J 99: 457–461. 10.1038/sj.bdj.4812743 16215581

[72] Davies HT, Crombie IK, Tavakoli M (1998) When can odds ratios mislead? BMJ 316: 989–991. 9550961 10.1136/bmj.316.7136.989 PMC1112884

[73] Petersen MR, Deddens JÁ (2008) A comparison of two methods for estimating prevalence ratios. BMC Med Res Methodol 8: 9. 18307814 10.1186/1471-2288-8-9 PMC2292207

[74] Silva FG, Tavares-Neto J (2007) Avaliação dos prontuários médicos de hospitais de ensino do Brasil. Rev bras educ med 31: 113–126.

[75] Liberati A, Altman DG, Tetzlaff J, Mulrow C, Gotzsche PC, et al (2009) The PRISMA statement for reporting systematic reviews and meta-analyses of studies that evaluate health care interventions: explanation and elaboration. PLoS Med 6: e1000100. 19621070 10.1371/journal.pmed.1000100 PMC2707010

