# Supplementary material for: Risk Factors for Adverse Prognosis and Death in American Visceral Leishmaniasis: A Meta-analysis
Source: PLoS Negl Trop Dis. 2014 Jul 24;8(7):e2982. doi: 10.1371/journal.pntd.0002982 (PMC4109848; doi:10.1371/journal.pntd.0002982)
Supplement: Table S1 — Main characteristics of the studies included in the systematic review on prognostic factors relating to visceral leishmaniasis (VL) severity. (DOC) [file pntd.0002982.s001.doc]

Table S1. Main characteristics of the studies included in the systematic review on prognostic factors relating to visceral leishmaniasis (VL) severity

| **Authors** | **Location** | **Population** | **Statistics/Control of confounding factors** | **Variables included** | **Outcome** |
| --- | --- | --- | --- | --- | --- |
| Werneck et al. [11] | Hospital of Infectious Diseases, Teresina, PI | Source of data: medical records  No. of individuals: 90 adults/children  Period: 18 months (years not specified) | Logistic regression (*P* = 0.10 and 0.05 for initial and final models, respectively) | Gender, age, duration of fever, cough, diarrhea, abdominal distension, hemorrhage, pallor, jaundice, hematocrit, leukocyte count, coinfection, history of blood transfusion, use of antibiotics, weight lost | Death |
| Sampaio et al. [18] | Hospital, Fortaleza, CE | Source of data: medical records  No. of individuals: 546 children/adolescents (≤ 14 years old)  Period: May 1996 - June 2006 | Logistic regression (*P* = 0.20 and 0.05 for initial and final models, respectively) | Gender, age, hemorrhage, bacterial infection, severe leukopenia, neutropenia, severe anemia, thrombocytopenia, nutritional status | Death |
| Alvarenga et al. [27] | University Hospital, Campo Grande, MS | Source of data: medical records and the Information System on Disease Notification (*Sistema de Informação de Agravos de Notificação;* SINAN)  No. of individuals: 76 adults  Period: 2002-2005 | Survival analysis without control of confounding factors | Gender, interval between onset of symptoms and diagnosis, comorbidities (liver and kidney diseases, HIV/AIDS and others conjointly analyzed) | Death |
| Araújo [28] | Municipality of Belo Horizonte, MG | Source of data: SINAN (with verification of data in the Municipal Secretary of Health, Belo Horizonte, MG)  Versions of SINAN: Windows (up to 2006) and Net (up to 2009)  No. of individuals: 546 adults / 342 children (0-10 years)  Period: 2002-2009 | Logistic regression (*P* = 0.25 and 0.05 for initial and final models, respectively). Two models were evaluated: (i) considering the combination of common variables during the period; (ii) considering the common variables together with those included in the Net system (2007- 2009) used by SINAN | Gender, age, interval between symptoms and notification, fever, splenomegaly, hepatomegaly, weakness, weight loss, cough, edema, pallor, coinfection (HIV/tuberculosis), bacterial infection, hemorrhage, jaundice, other clinical manifestations, initial therapy | Death |
| Braga [29] | Pediatric Hospital, Belo Horizonte, MG | Source of data: medical records  No. of individuals: 250 children (< 12 years old)  Period: 2001-2005 | χ^2^ or Fisher exact tests and estimation of the Relative risk for the categorical variables. For continuous data: Student *t* test (variables with normal distribution) or Kruskal Wallis (non-parametric variables) | Interval between onset of symptoms and hospital admission, beginning of symptoms, prostration, age, weight loss, reduced appetite, abdominal distension, cough, jaundice, edema, abdominal pain, hemorrhage, vomiting, diarrhea, pallor, cardiovascular and respiratory abnormalities, dehydration, poor perfusion, liver and spleen sizes, hemoglobin, mean cell volume, mean cell hemoglobin, neutrophil, leukocyte and platelet counts, urea, creatinine, aspartate transaminase (AST), alanine transaminase (ALT), globulin, albumin | Favorable or unfavorable evolution (sepsis, pneumonia, urinary infection, and/or any type of hemorrhage, except epistaxis, and/or total neutrophil count < 500 neutrophils/mm^3^) |
|  |  |  |  |  |  |
| Cavalcante [30] | Pediatric Hospital, Fortaleza, CE | Source of data: follow up of an open cohort  No. of individuals: 75 children/adolescents (3 months - 19 years old)  Period: June 2005 - August 2006 | Wilcoxon rank sum and χ^2^ tests or Fisher exact test. Wilks**'** lambda distribution and standard discriminant analysis to develop a predictive model for clinical cure up to the 20^th^ day of treatment | Heart rate, liver and spleen sizes, interval between onset of fever and hospital admission, hemoglobin concentration, hematocrit, leukocyte, neutrophil, lymphocyte, eosinophil and platelet counts, hemo-sedimentation rate, levels of albumin and globulin, prothrombin time, AST and ALT levels, pallor, jaundice, edema, hemorrhage, fever, coinfection, pneumonia or sepsis, duration of fever, age, nutritional status | Two outcomes: (i) Favorable (disappearance of fever, weight gain, 50% reduction in spleen size, satisfactory hematological markers) or unfavorable prognosis until the 20^th^ day; (ii) recovery or death |
| Costa et al [31] | Hospital of Infectious Diseases, Teresina, PI | Source of data: medical records  No. of individuals: 179 adults/ 216 children (0-15 years)  Period: 1996 - 2005 | Logistic regression (*P* = 0.05 for all models) | Age, undernutrition, vomiting, diarrhea, dyspnoea, edema, hemorrhage, pulmonary rattling, severe anemia, severe neutropenia, severe thrombocytopenia, hepatic lesion (ALT > standard value), serum albumin, kidney lesion, proteinuria, parasite load in the medulla (high or low), previous hemorrhage (petechiae), bacterial infection, HIV | Death |
| Costa [32] | Institute of Tropical Diseases Natan Portela, Teresina, PI | Source of data: follow up of an open cohort  No. of individuals: 420 adults/ 463 children (0-5 years)  Period: September 2005 - August 2008 | Logistic regression (*P* = 0.20 and 0.05 for initial and final models, respectively) | Age, hemorrhage (1, 2-3 or ≥ 4 sites), petechiae, ecchymoses, platelet count, hemoglobin concentration, AST, ALT and alkaline phosphatase levels, bilirubin, neutrophil and lymphocyte counts, kidney failure, chest x-ray (normal, alveolar and interstitial opacity), HIV coinfection, adynamia, drowsiness, vomiting, inappetence, diarrhea, constipation, abdominal pain, cough, dyspnoea, edema, bacterial infections, hepatomegaly, splenomegaly, fever (< 39°C) | Death |
| Madalosso [33] | In all cases, the probable location of VL infection was the State of São Paulo. Health units at which the patients were admitted were also mentioned | Source of data: medical records  No. of individuals: 376 of 559 confirmed VL cases : 174 adults/ 202 children (0-10 years) of autochthonous transmission in the State of São Paulo, according to SINAN.  Period: 1999 - 2005 | Logistic regression (*P* = 0.05 for all models) | Gender, age, fever, asthenia, weight loss, dry cough, abdominal distension, abdominal pain, anorexia, diarrhea, vomiting, splenomegaly, hepatomegaly, pallor, manifestations of hemorrhage (epistaxis, petechiae, melena, bleeding gums), dyspnoea, pulmonary abnormalities, edema, jaundice, adenomegaly, undernutrition, serum albumin, total bilirubin, hematocrit, hemoglobin, anemia, platelet, leukocyte and neutrophil counts, AST and ALT levels, interval between onset of symptoms and diagnosis, recurrence interval, comorbidities (AIDS), peripheral vascular disease, diabetes mellitus with or without complications, congestive heart failure, moderate/severe liver disease, kidney disease, tuberculosis, chronic obstructive pulmonary disease, splenectomy, leukemia, lymphoma, altered chest x-ray, altered electrocardiogram, bacterial infections and/or sepsis | Death |
| Oliveira [34] | Nineteen hospitals, Belo Horizonte, MG; One University Hospital, Montes Claros, MG | Source of data: medical records  No. of individuals: 50 adults/ 387 children  Period: July 1999 - December 2000 (Belo Horizonte) and January 1999 – December 2002 (Montes Claros) | Population divided into two groups (< 15 and ≥ 15 years old) with separate analysis. Logistic regression (*P* = 0.25 and 0.05 for initial and final models, respectively) | Gender, age, duration of disease, jaundice, edema, liver and spleen size, AST and ALT levels, hematocrit, hemoglobin concentration, leukocyte and platelet counts | Death |
| Queiroz [35] | University Hospital, Recife, PE | Source of data: medical records  No. of individuals: 431 children/adolescents (≤ 14 years old)  Period: May 1996 - December 2001 | Logistic regression (*P* = 0.20 and 0.05 for initial and final models, respectively) | Gender, age, duration of disease, undernutrition (moderate/severe; slight/eutrophic), edema, hemorrhage, jaundice, dyspnoea, spleen size, infection, previous therapy against VL, hemoglobin concentration, leukocyte, neutrophil and platelet counts | Death |
|  |  |  |  |  |  |
| Rey et al. [36] | Pediatric Hospital, Fortaleza, CE | Source of data: medical records  No. of individuals: 450 children  Period: 1995 - 2002 | Student *t* and χ^2^ tests for continuous and categorical variables, respectively. Odds ratio stratified according to age, jaundice, hemorrhage, edema and bacterial infection | Gender, age, nutritional status (well nourished or moderately well nourished *vs.* moderately undernourished or severely undernourished), fever, pallor, adnominal distension, anorexia, weakness, weight loss, edema, hemorrhage, jaundice, splenomegaly, hepatomegaly, hemoglobin concentration, leukocyte, neutrophil, platelet and erythrocyte counts, prothrombin activity, serum globulin, nosocomial infection, diarrhea, lack of air | Death |
| Santos et al. [37] | Hospital of Infectious Diseases, Teresina, PI | Source of data: medical records and interviews with family members  No. of individuals: 116 adults/ 64 children (0-10 years) treated with amphotericin B interned for a period of  two years  Period: (years not specified) | χ^2^ or Mann-Whitney tests initially; and model adjusted subsequently by logistic regression | Gender, age, duration of fever, weight loss, cough, diarrhea, abdominal distension, hemorrhage, edema, pallor, jaundice, pulmonary rattling, spleen size, hematocrit, leukocyte count, presence of parasites in the medulla, coinfection | Amphotericin B treatment; persistence of signs and symptoms (mainly splenomegaly) after administration of antimonials or recurrences |
| Souza [38] | Hospital Eduardo de Menezes and outpatient clinic of the Reference Center for Leishmaniasis of FIOCRUZ, Belo Horizonte, MG | Source of data: medical records  No. of individuals: 65 adults. Period: January 2000 – December 2005 | Logistic regression (*P* = 0.25 and 0.05 for initial and final models, respectively) | Gender, edema, fever, cough, HIV coinfection, abdominal pain, jaundice, evolution time (> 8 weeks), diarrhea, hepatomegaly, splenomegaly, lymphadenopathy, hemorrhage, anemia, leukopenia, lymphocytopenia, thrombocytopenia. | Total recovery or not observed 15 days after treatment (resolution of all signs and symptoms investigated) |
