# Supplementary material for: Risk Factors for Adverse Prognosis and Death in American Visceral Leishmaniasis: A Meta-analysis
Source: PLoS Negl Trop Dis. 2014 Jul 24;8(7):e2982. doi: 10.1371/journal.pntd.0002982 (PMC4109848; doi:10.1371/journal.pntd.0002982)
Supplement: Table S2 — Predictors, classified according to strength, of unfavorable clinical evolution independent of death and mortality for American visceral leishmaniasis identified in this systematic review. (DOC) [file pntd.0002982.s002.doc]

**Table S2**. Predictors, classified according to strength, of unfavorable clinical evolution independent of death and mortality for American visceral leishmaniasis identified in this systematic review

| Group | Predictor | Summary measures  Odds Ratio (OR) or *P*-values (95% CI)  Outcome: death* | I^2^ test/Data pattern | Control of confounding factors | Results for unfavorable clinical evolution independent of death |
| --- | --- | --- | --- | --- | --- |
| I. Significant summary measures ordered according to strength; consistent pattern towards clinical evolution and mortality; significance in the majority of multivariate analyses | Jaundice | 6.3 (3.9 - 10.3)  [11, 18, 28, 30 – 36] | 56.81  All studies had similar pattern | Significance in eight of the ten studies that controlled confounding factors | Considered a risk factor in two (OR = 7,5; 1,4 - 39,4 and 5,2; 0,2 – 110,8) [29, 30] of the three studies (OR = 0,7; 0,2 – 2,5) [29, 30, 38] that had information available (*P* < 0.001) |
|  | Thrombocytopenia (cut-off points: < 50,000 and/or < 10,000 platelets/mm^3^) | 5.5 (3.0 – 10.3)  [18, 31 – 34, 36] | 70.83  All studies had similar pattern | Significance in four of the six studies that controlled confounding factors | Considered a risk factor in studies with cut-off point < 50,000 (OR = 4,9; 2,6 – 9,4) [29] and 150,000 (OR = 1,3; 0,4 – 4,8) [38] platelets/mm^3^. The mean platelet count was higher in cured individuals (difference in means = 32,921) [30] |
|  | Hemorrhage | 5.0 (3.5 – 7.2)  [18, 28, 30 – 33, 35, 36] | 27.83  All studies had similar pattern | Significance in four of the six studies that controlled confounding factors | Considered a risk factor in the two studies (OR = 2,1; 0,2 – 24,3 and 16,3; 3,5 – 24,3) [29, 30] that had information available (*P* < 0.001) |
|  | HIV Coinfection | 3.7 (2.1 – 3.6)  [28, 31, 32, 38] | 16.47  All studies had similar pattern | Significance in the three studies that controlled confounding factors | Considered a risk factor in the only study that had information available (OR = 9,9; 2,0 – 49,1) [38] |
|  | Diarrhea | 2.8 (2.0 – 3.8)  [11, 18, 31 – 33] | 0 | Significance in three of the five studies that controlled confounding factors | Considered a risk factor in the two studies (OR = 1,8; 0,8 – 4,1 and 2,6; 0,6 – 10,6) [29, 38] that had information available (*P* = 0.04) |
|  | Age (*≤* 1 year *vs.* > 1-19 years) | 2.7 (1.6 – 4.7)  [28, 30 – 33, 36] | 13.93  All studies had similar pattern | Different comparisons: (i) all ages *vs.* ≤ 1 or 5 years, (ii) ≤ 5 years *vs* > 5-19 years, and (iii) variable considered continuous [34]. Significance in three of the five studies that controlled confounding factors | Greater risk for children < 6 (OR = 2,1; 0,5 – 10,0), 12 (OR = 2,4; 1,1 – 5,7) and 18 months (OR = 3,1; 1,6 – 6,0) compared with children ≤ 6 years [29]; greater risk for children < 2 years compared with children ≤ 15 years (OR = 1,8; 0,6 – 6,0) [30]; greater risk for children 1-10 years compared with adults (OR = 10,2; 2,2 – 47,1) [37] |
|  | Age (≤ 5 years *vs.* > 5-19 years) | 2.39 (1.40 – 4.10)  [18, 28, 30, 31, 33, 35] | 26.24  All studies had similar pattern |  |  |
|  | Severe neutropenia (< 500 neutrophils/mm^3^ | 2.3 (1.5 – 3.6)  [18, 31, 32, 38] | 40.77  All studies had similar pattern | Significance in three out of the four studies that controlled confounding factors | Mean neutrophil count was low in individuals with unfavorable evolution (diference in means = 546) [30]; |
|  |  |  |  |  |  |
| II. Significant summary measures ordered according to strength; consistent pattern towards mortality; no studies relating exclusively to clinical evolution; significance in the majority of multivariate analyses | Age (> 50 years *vs.* other age groups) | 3.9 (2.6 – 5.7)  [28, 33] | 96.47  All studies had similar pattern | Significance in all comparisons performed: (i) > 40, > 50 and > 60 years *vs.* other age groups; (ii) > 40 years *vs.* 1-20 years; (iii) > 25 years *vs.* ≤ 25 years (only individual > 15 years) [34] | Information was not available in any study |
|  | Age (> 40 years *vs.* other age groups) | 3.1 (2.3 – 4.3)  [28, 31 – 33] | 90.77  All studies had similar pattern |  |  |
|  | Dyspnoea | 3.2 (2.3 – 4.6)  [18, 31 – 33, 35] | 0 | Significance in four of the five studies that controlled confounding factors | Information was not available in any study |
|  | Bacterial coinfections | 3.1 (2.4 – 4.1)  [18, 28, 31 – 33, 35, 36] | 0 | Significance in four of the seven studies that controlled confounding factors | Information was not available in any study |
| III. Significant summary measures ordered according to strength; consistent pattern towards clinical evolution and mortality; no significance in the majority of multivariate analyses | Edema | 4.0 (3.0 – 5.3)  [18, 28, 30 – 36] | All studies had similar pattern | Significance in four of the eight studies that controlled confounding factors | Considered a risk factor in two studies (OR = 4,2; 1,9 – 9,6 and 2,3; 0,7 – 8,0) [29, 30] and a protection factor in one study (OR = 0,4; 0,1 – 3,4) [38] |
|  | Hemoglobin (< 7 or 5 g/dL) | 3.1 (2.1 – 4.6)  [18, 31 – 35] | 46.37  All studies had similar pattern | Significance in two of the seven studies that controlled confounding factors | Considered a risk factor in the two studies (cut-off point = 11g/dL: OR = 11,1; 1,1 – 108,4 and cut-off point = 7g/dL: OR = 3,1; 1,7 – 5,5) [29, 38] that had information available (*P* < 0.001) |
|  | Vomiting | 2.5 (1.7 – 3.7)  [31 - 33] | 29.6  All studies had similar pattern | Significance in one of the three studies that controlled confounding factors | Considered a risk factor in the study (OR = 1,1; 0,5 – 2,0) [29] that had information available |
|  | Alanine transaminase (ALT > defined cut-off point) | *P* < 0.001  [31, 32, 34] | All studies had similar pattern | Significance in one of the four studies that controlled confounding factors | Considered a risk factor in a study (OR = 3,7; 1,5 – 8,7) [29] with cut-off point of 105 mg/dL. Mean ALT levels (difference = 35mg/dL) were higher in individuals with adverse evolution [30] |

|  | Undernutrition (weight-for-age < defined cut-off point | *P* < 0.001  [18, 30, 31, 35, 36] | All studies had similar pattern | Significance in one of the four studies that controlled confounding factors | Considered a risk factor in the two studies (OR = 1,7; 0,7 – 1,1 and 1,8; 0,6 – 5,3) [29, 30] that had information available, although without statistical significance in meta-analysis (*P* = 0.09) |
| --- | --- | --- | --- | --- | --- |
|  | Aspartate transaminase (AST; level > defined cut-off point) | *P* < 0.001  [32 - 34] | All studies had similar pattern | Significance in one of the four studies that controlled confounding factors | Considered a risk factor in a study (OR =1,8; 1,0 – 3,2) [29] with cut-off point of 110 mg/dL. Mean AST levels were higher in individuals with adverse evolution (difference in means = 72 mg/dL) [30] |

| IV. Significant summary measures ordered according to strength; different patterns towards clinical evolution; no significance in the majority of multivariate analyses | Interval between the onset of fever and diagnosis (> 48 or > 60 days; results only for adults)  Interval between the onset of fever and diagnosis (> 21 or > 60 days; results only for children) | 1.9 (1.2 – 2.9)  [11, 28, 33, 34]  0.42 (0.20 – 0.80)  [30, 34, 35] | 63.32  All studies had similar pattern  All studies had similar pattern (protection factor) | Significance in two of the four studies that controlled confounding factors  Significance in one of the three studies that controlled confounding factors | Considered a protective factor in one study [38] that had information available (cut-off point 32 days: OR = 0,7; 0,2 – 2,2)  Two studies (cut-off point 50 days: OR = 8; 2,2 – 29,2 and cut-off point 20 days: OR = 1,8; 1,0 – 3,2)  [29, 30] identified the variable as a risk factor |
| --- | --- | --- | --- | --- | --- |
|  | Hematocrit (< defined cut-off point) | *P* = 0.03  [11, 34] | Two studies had similar pattern and one was different | Significance (risk factor) in one of the three studies that controlled confounding factors | No difference in means between cured and non-cured individuals in one study [30] |
|  |  |  |  |  |  |
| V. Significant summary measures ordered according to strength; consistent pattern towards clinical evolution and mortality; no significance in all multivariate analyses | Pulmonary rales | 3.4 (2.3 – 5.0)  [31, 32] | All studies had similar pattern | No significance in the two studies that controlled confounding factors | Considered a risk factor in the only study available (OR = 8,0; 2,4 – 26,6) [29] |
|  | Serum albumin (< 3.0 g/dL) | 3.0 (1.7 – 5.1)  [31, 33] | All studies had similar pattern | No significance in the two studies that controlled confounding factors | Considered a risk factor in the two studies available: (i) compared mean values (difference in means = 0,6 g/dL) [30]; (ii) used cut-off point of 2.5 g/dL (OR = 10,3; 4,0 – 26,3) [29] |
|  | Pallor | 2.1 (1.2 – 3.6)  [28, 30, 33] | All studies had similar pattern | No significance in the three studies that controlled confounding factors | Considered a risk factor in two studies (OR = 5,4; 0,7 – 41,5 and 2,1; 0,2 – 24,3) [29, 30] (*P* = 0.038) |
|  | Cough | 1.7 (1.3 – 2.3)  [28, 32, 33] | All studies had similar pattern | No significance in the three studies that controlled confounding factors | Considered a risk factor in the two studies (OR = 1,7; 0,9 – 3,2 and 1,9; 0,6 – 6,0) [29, 38] (*P* < 0.001) |
|  |  |  |  |  |  |
| VI. Other weak predictors | Drowsiness | 3.5 (2.2 – 5.4)  [32, 33] | All studies had similar pattern | No significance in the two studies that controlled confounding factors | Information not available in any study |
|  | Altered thorax X-ray | 3.3 (2.0 – 5.6)  [32, 33] | All studies had similar pattern | No significance in the two studies that controlled confounding factors | Information not available in any study |
|  | Gender (results for children; referenced to females, OR = 1.0) | 0.5 (0.3 – 0.7)  [18, 34, 35] | All studies had similar pattern | No significance in the three studies that controlled confounding factors | Information not available in any study |
|  | Gender (results for adults; referenced to females, OR = 1.0) | 1.6 (1.0 – 2.4)  [11, 28, 34] | All studies had similar pattern | No significance in the two studies that controlled confounding factors | One study [38] with pattern opposite to that observed for lethal outcome (OR = 0,5; 0,1 – 2,5). |
|  | Fever | 0.4 (0.2 – 0.6)  [28, 30, 32, 33] | All studies had similar pattern | No significance in the three studies that controlled confounding factors | One study considered fever a protective factor (OR = 0,3; 0,03 – 3,2) [30], while another [38] considered variable a risk factor (OR = 1,1; 0,1 – 12,5) (*P* = 0.82) |
|  | Hepatomegaly (liver size > defined cut-off point) | 1.4 (1.0 – 2.2)  [28, 32 - 34] | All studies had similar pattern | No significance in the five studies that controlled confounding factors | Considered a risk factor in two studies that used cut-off points (OR = 2,1; 1,2 – 3,7 and 1,9; 0,4 – 8,1) [29, 38]. Mean size of liver was higher in individuals with adverse evolution (difference in means = 1 cm) [30] |
|  | Weight loss | 1.5 (1.0. – 2.2)  [28, 33] | All studies had similar pattern | No significance in the two studies that controlled confounding factors | One study (OR = 0,86; 0,44 – 1,6) [29] with opposite pattern |
|  | Splenomegaly (spleen size > defined cut-off point) | 1.2 (0.5 – 2.9)  [28, 32 - 35] | Four studies in the direction of protection and one in the direction of risk | Significance in one (with direction of protection) of the four studies that controlled confounding factors | Considered a risk factor in two studies that used cut-off points (OR = 1,6; 0,9 – 2,8 and 11,7; 2,6 – 52,2) [29, 38]. Mean size of liver was higher in individuals with adverse evolution (difference in means = 1 cm) [30] |

*Some studies were not included in meta-analysis procedures
