# Supplementary material for: Risk Factors for Adverse Prognosis and Death in American Visceral Leishmaniasis: A Meta-analysis
Source: PLoS Negl Trop Dis. 2014 Jul 24;8(7):e2982. doi: 10.1371/journal.pntd.0002982 (PMC4109848; doi:10.1371/journal.pntd.0002982)
Supplement: Text S1 — Forest plots for the variables submitted to meta-analysis. (DOCX) [file pntd.0002982.s003.docx]

**Text S1.** Forest plots for the variables submitted to meta-analysis

**I. Jaundice**

**
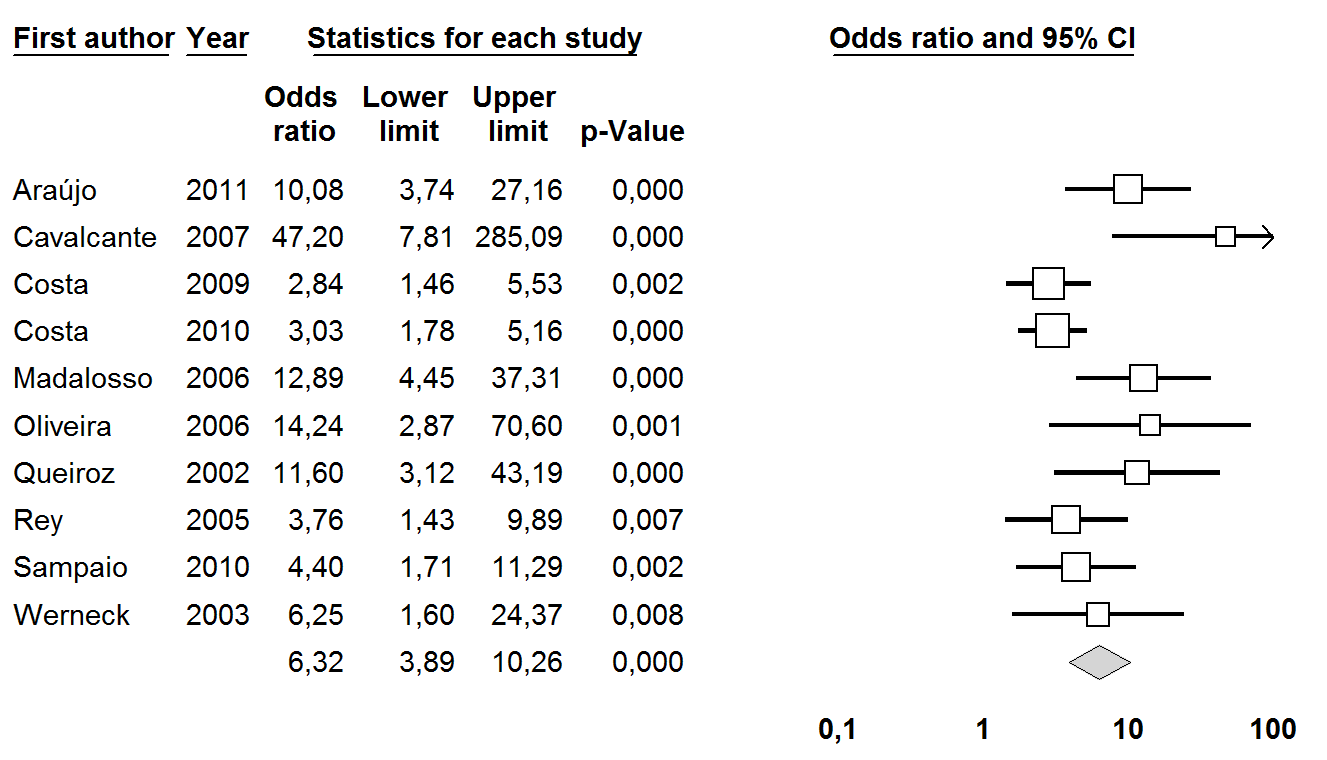
**

**II. Thrombocytopenia**

**
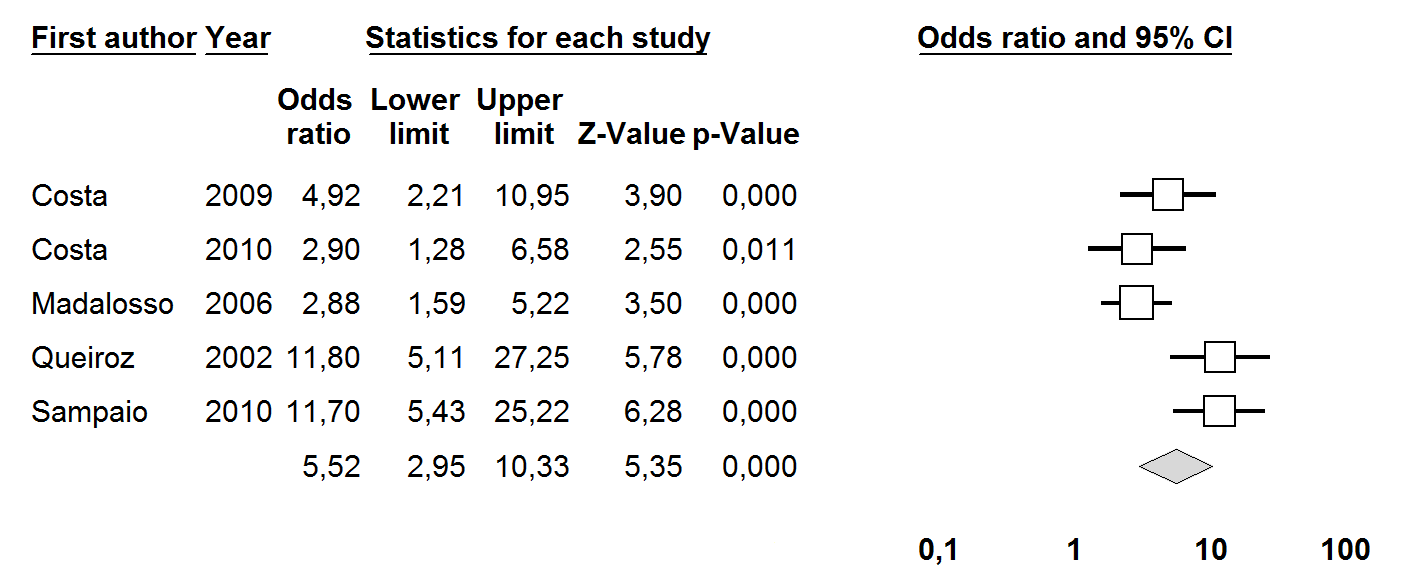
**

**III. Hemorrhage**

**
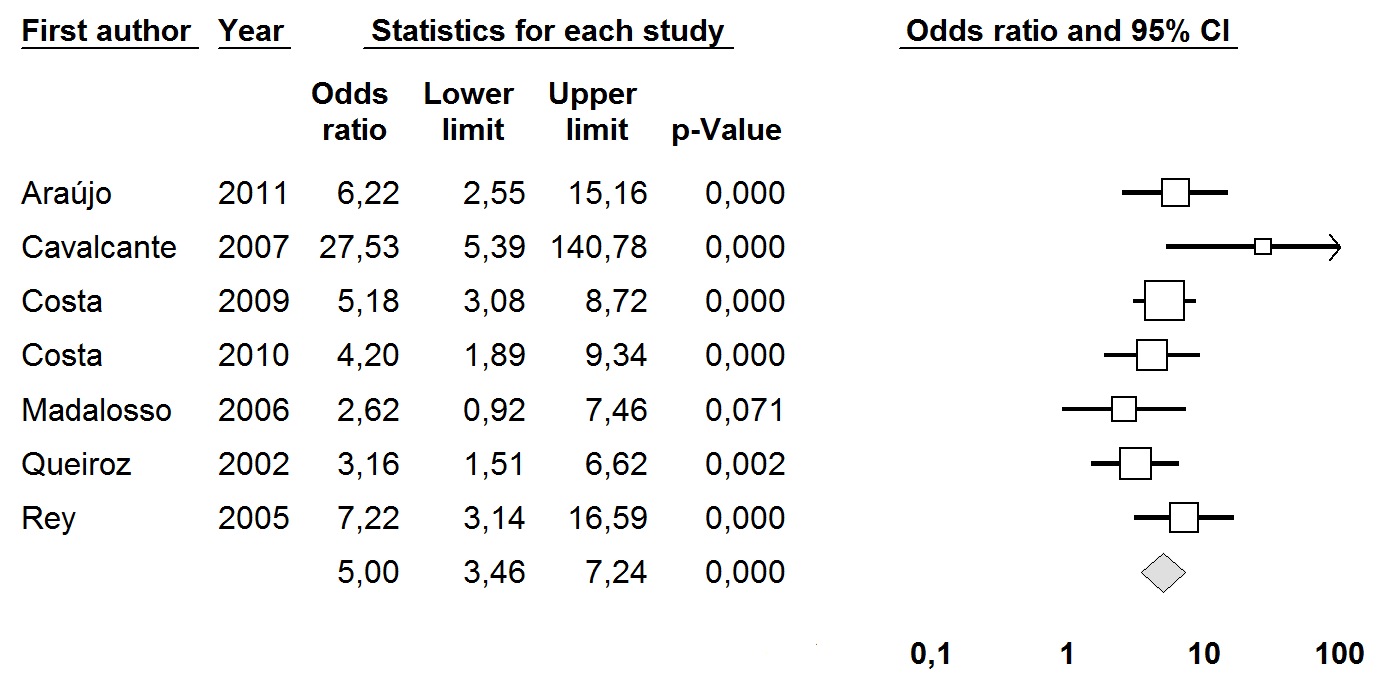
**

**IV. HIV Coinfection**

**
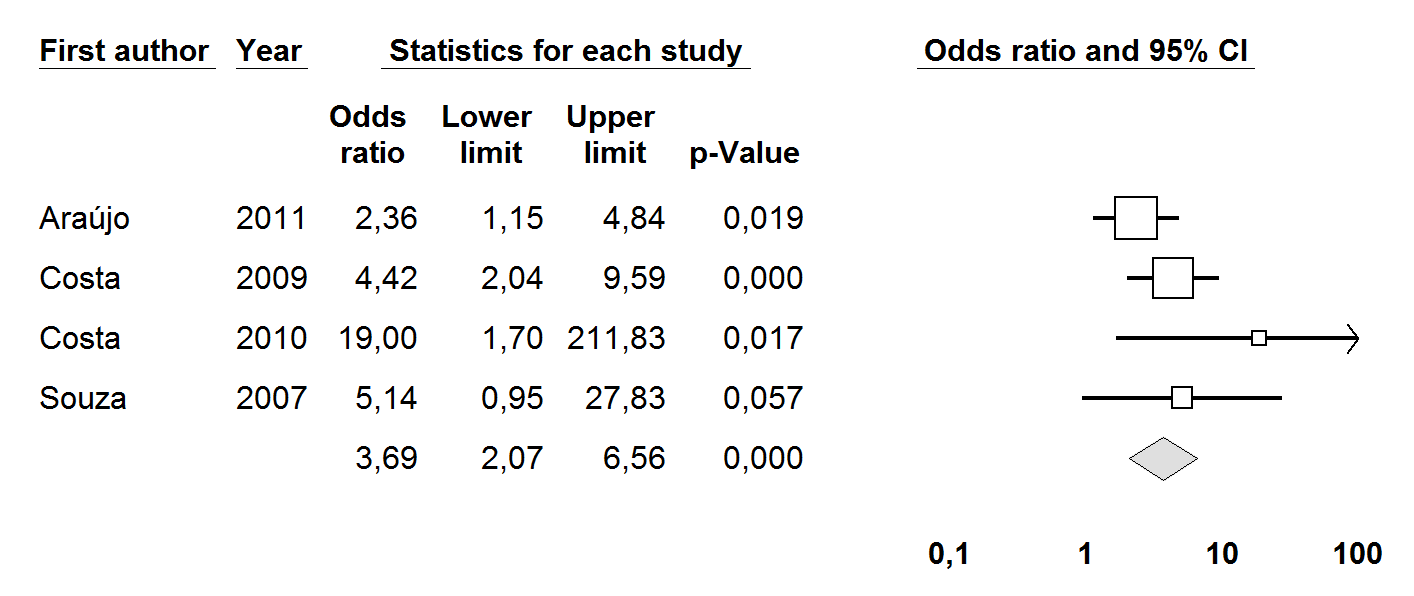
**

**V. Diarrhea**

**
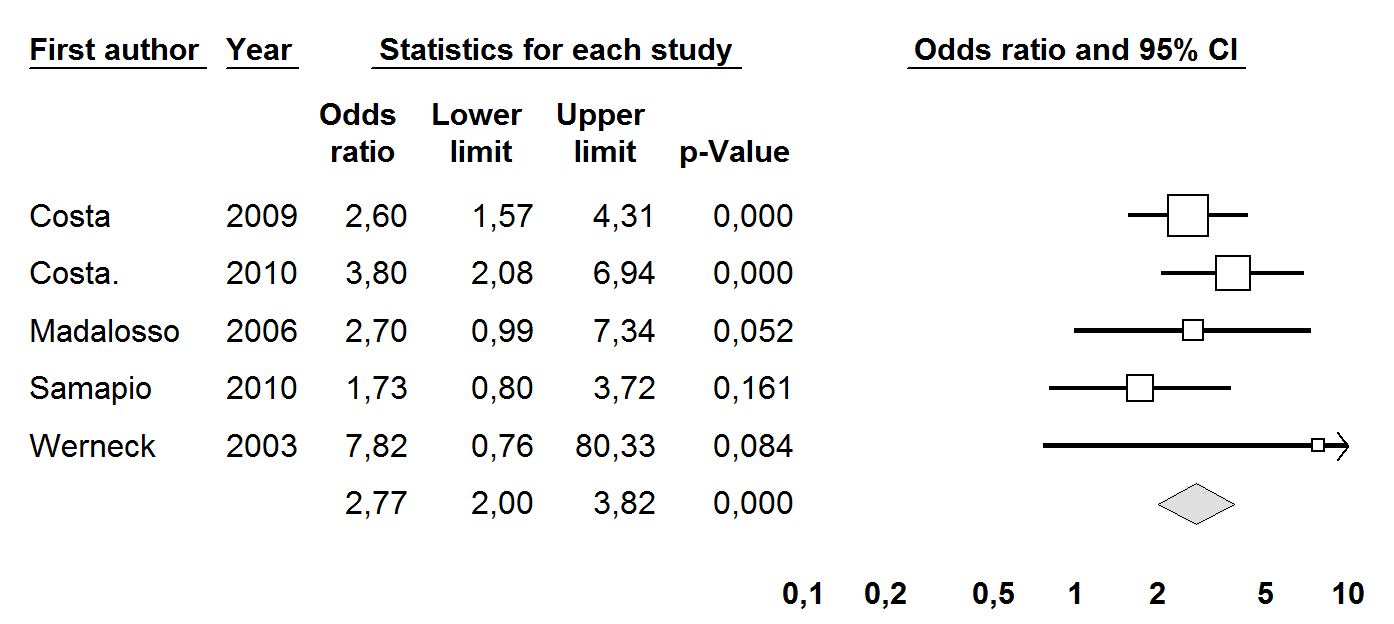
**

**VI. Age (≤ 1 year vs. > 1-19 years)**

**
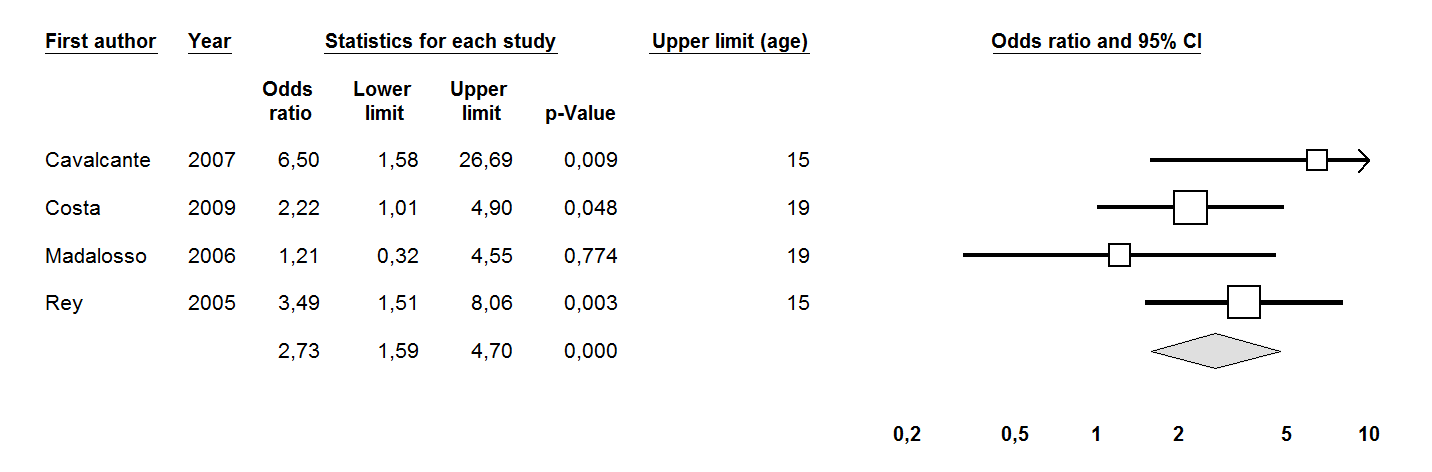
**

**VII. Age (≤ 5 years vs. > 5-19 years)**


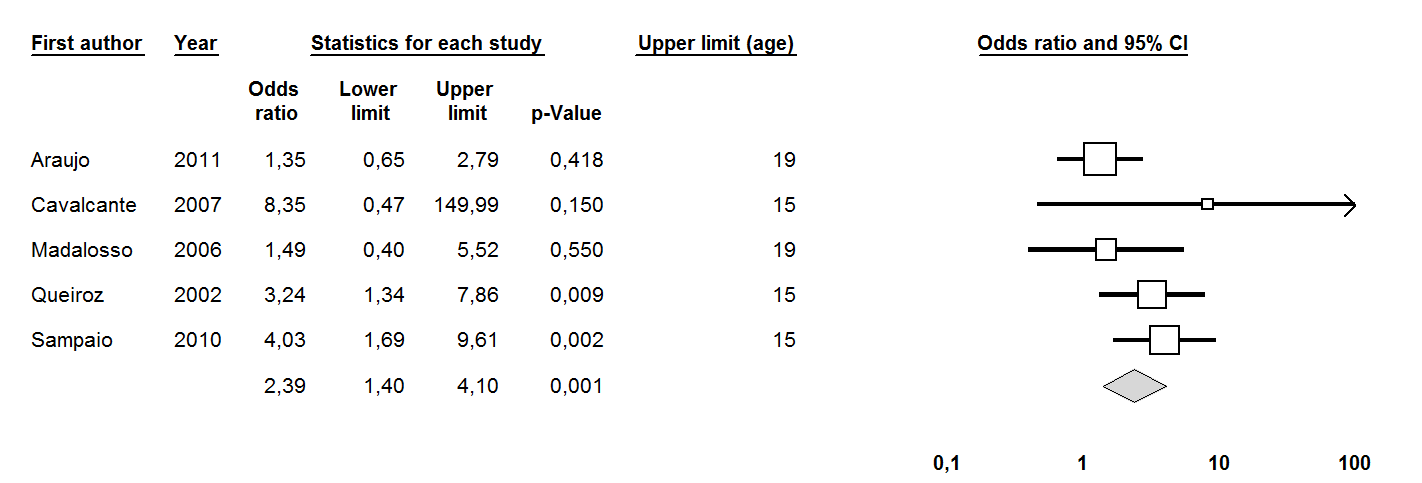


**VIII. Severe neutropenia (< 500 neutrophils/mm^3^)**

**
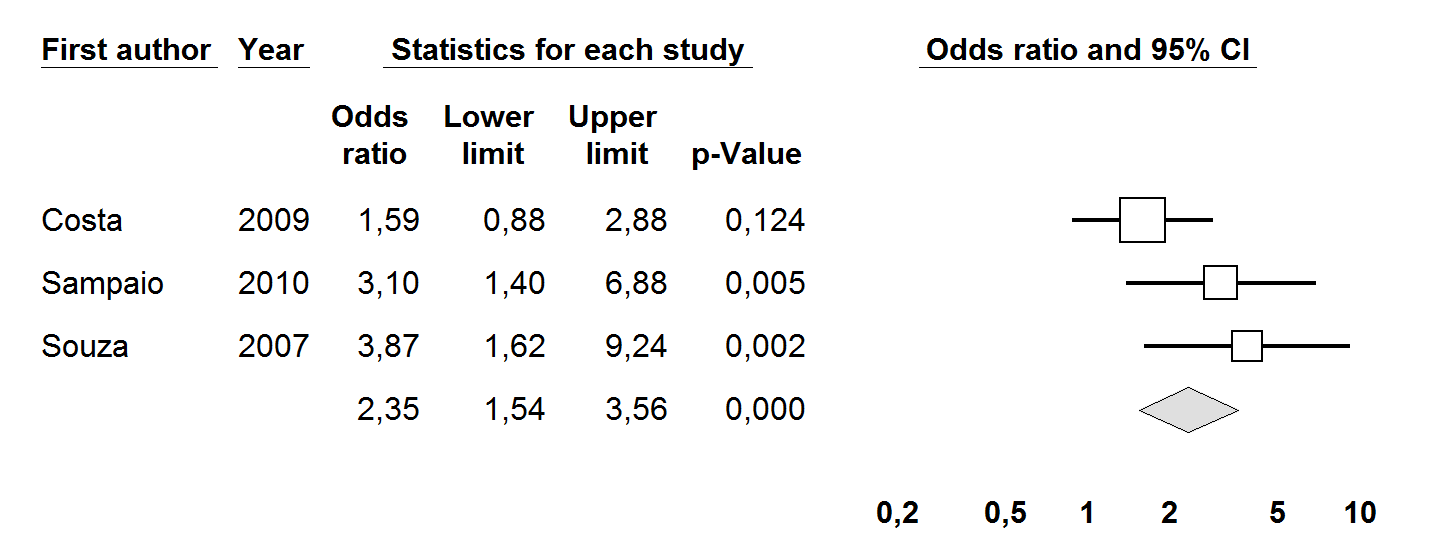
**

**IX. Age (> 50 years vs. other age groups)**

**
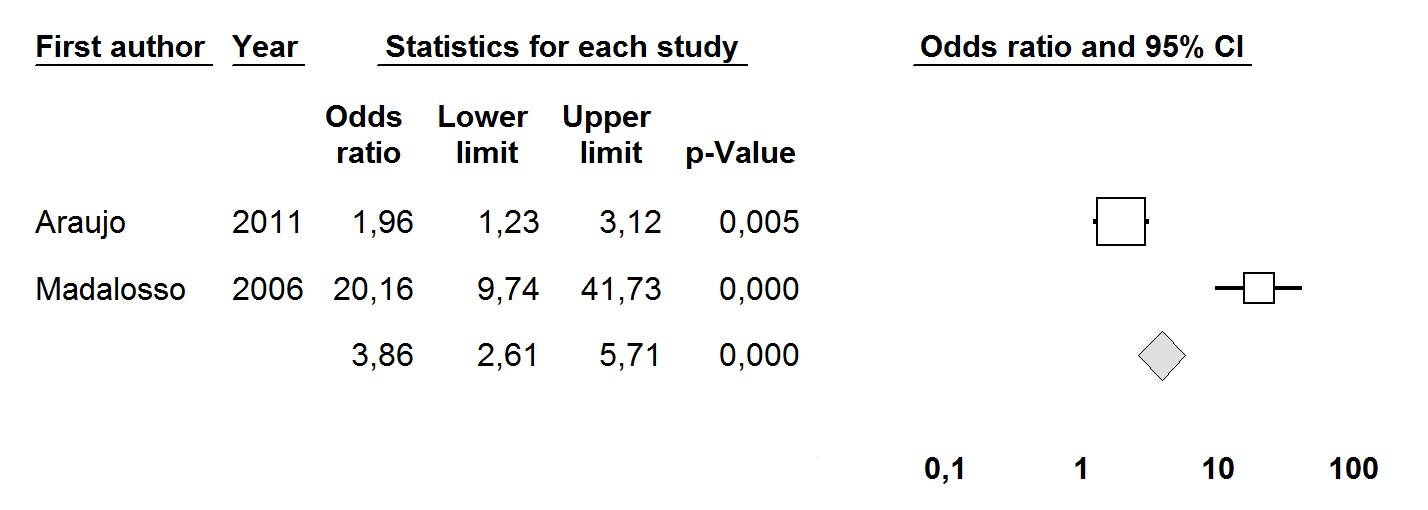
**

**X. Age (> 40 years vs. other age groups)**


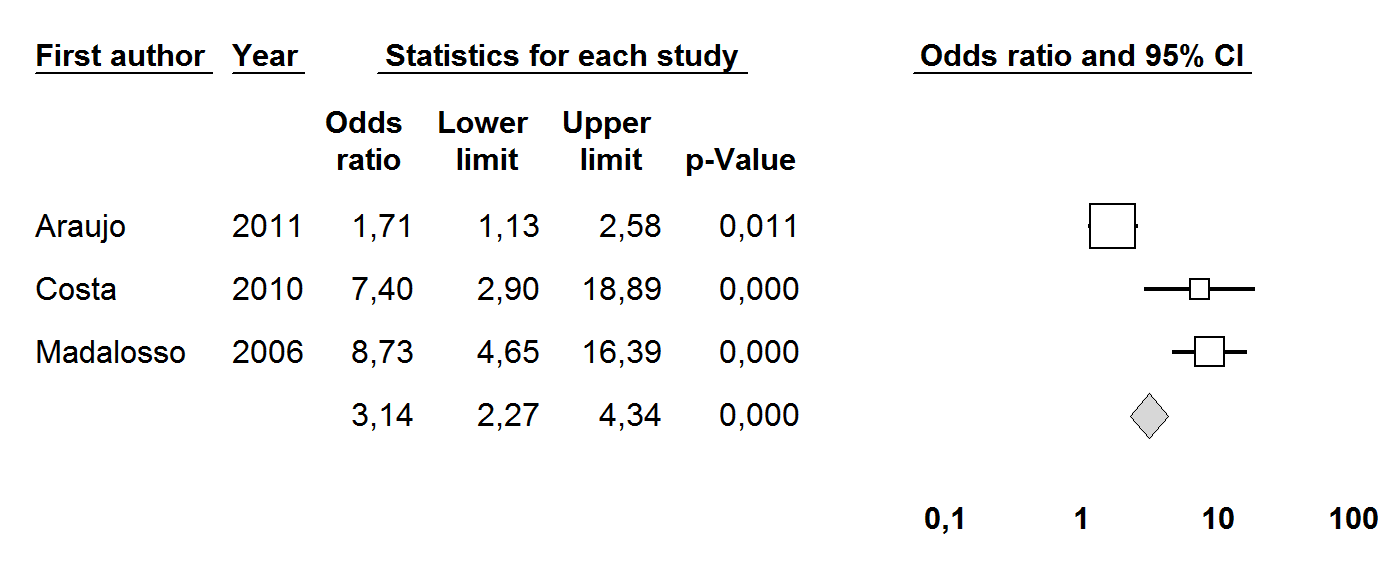


**XI. Dyspnoea**

**
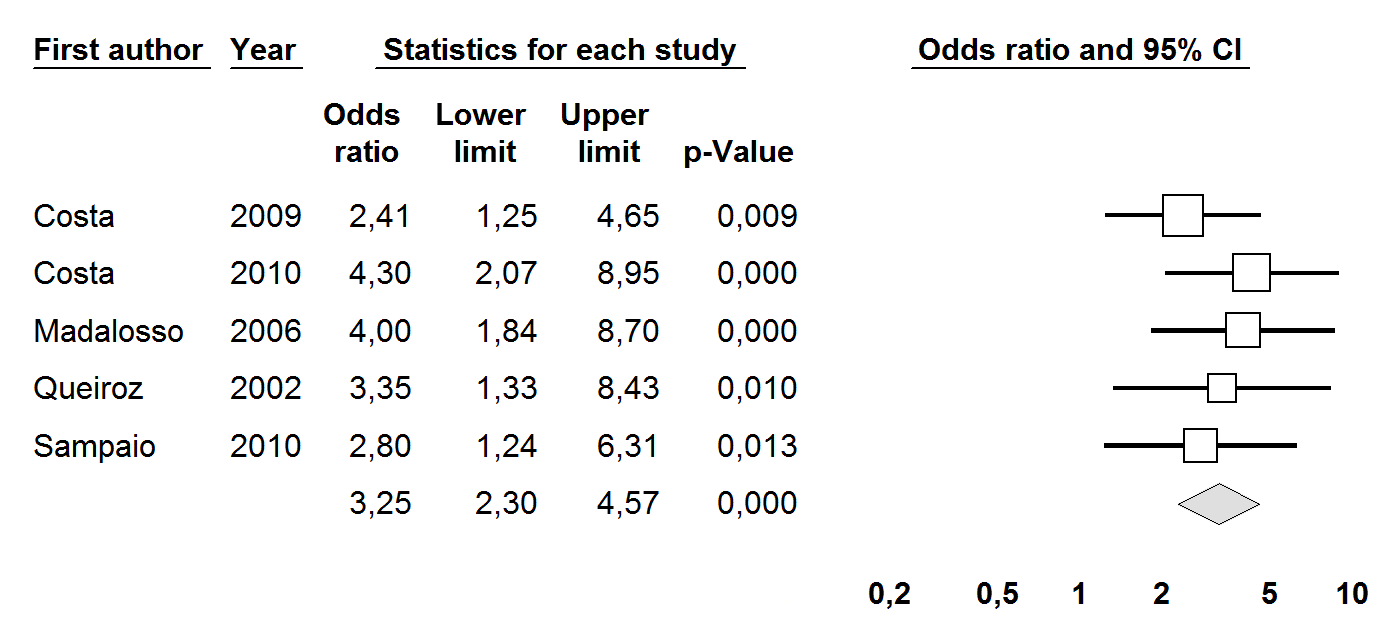
**

**XII. Bacterial coinfections**

**
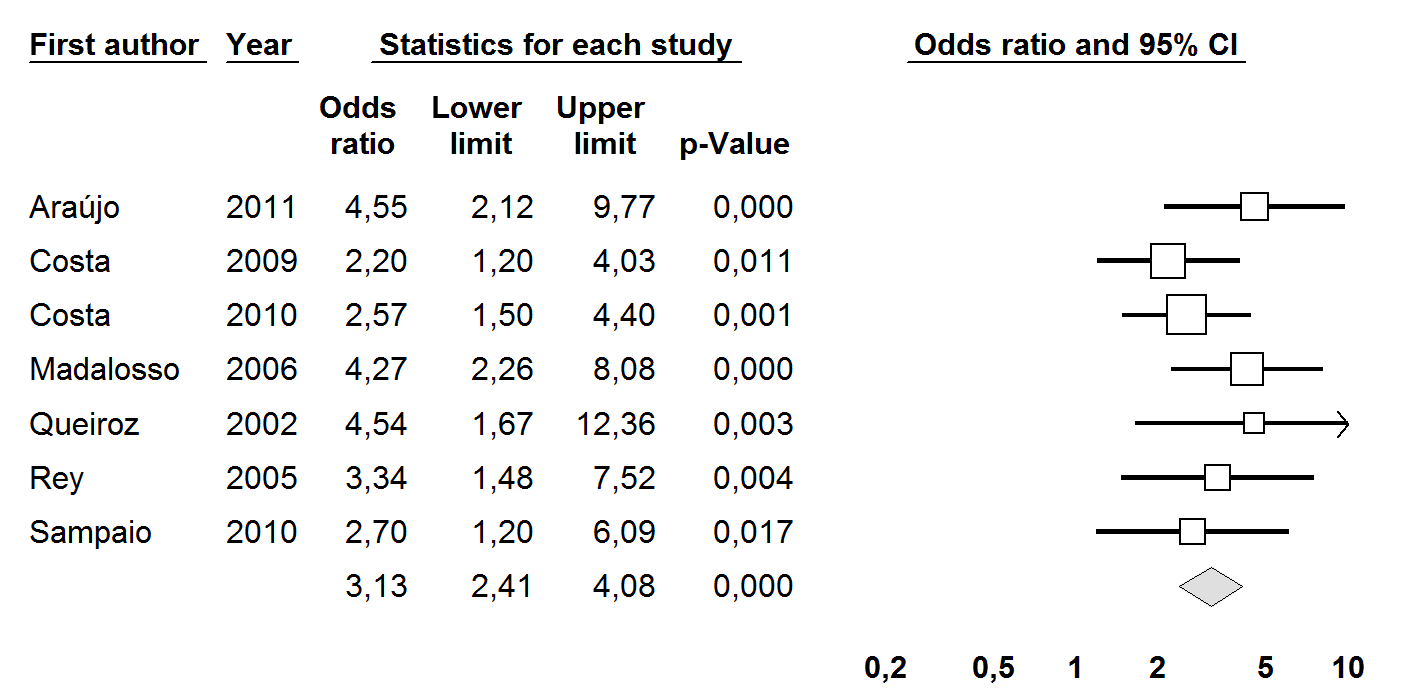
**

**XIII. Edema**

**
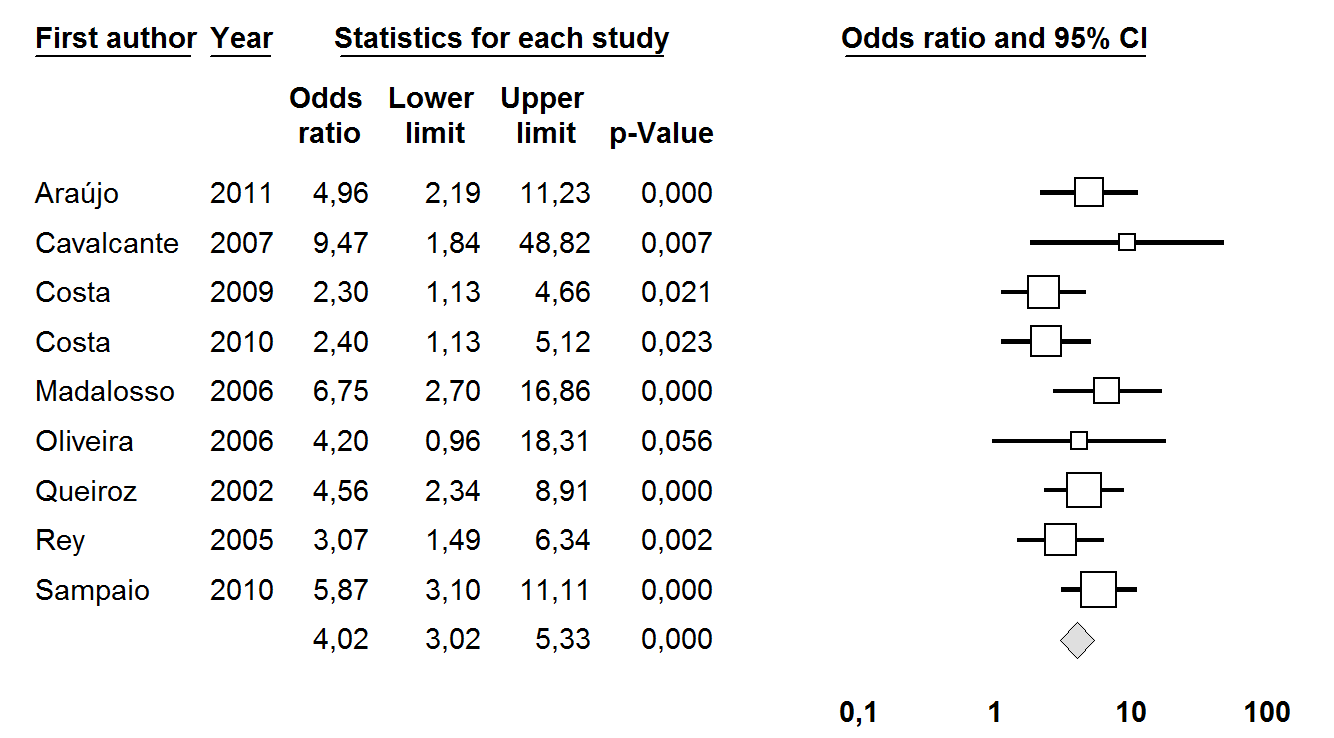
**

**XIV. Hemoglobin (< 7 or 5 g/dL)**

**
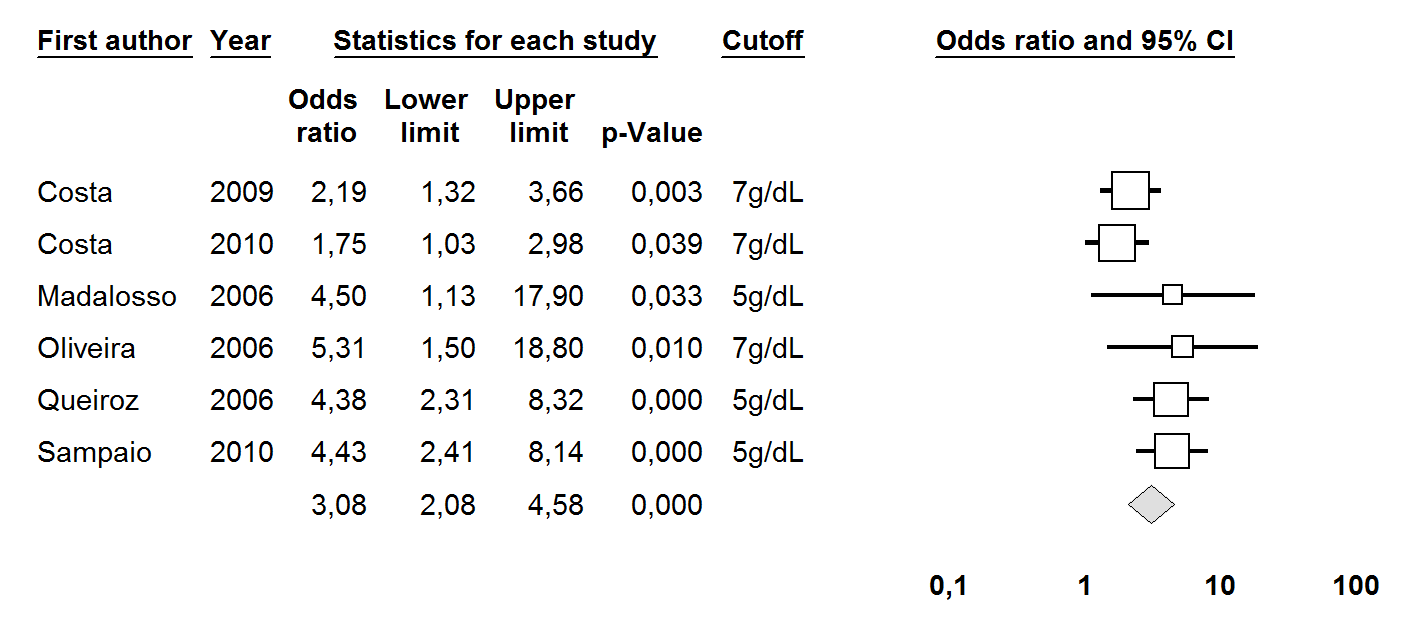
**

**XV. Vomiting**


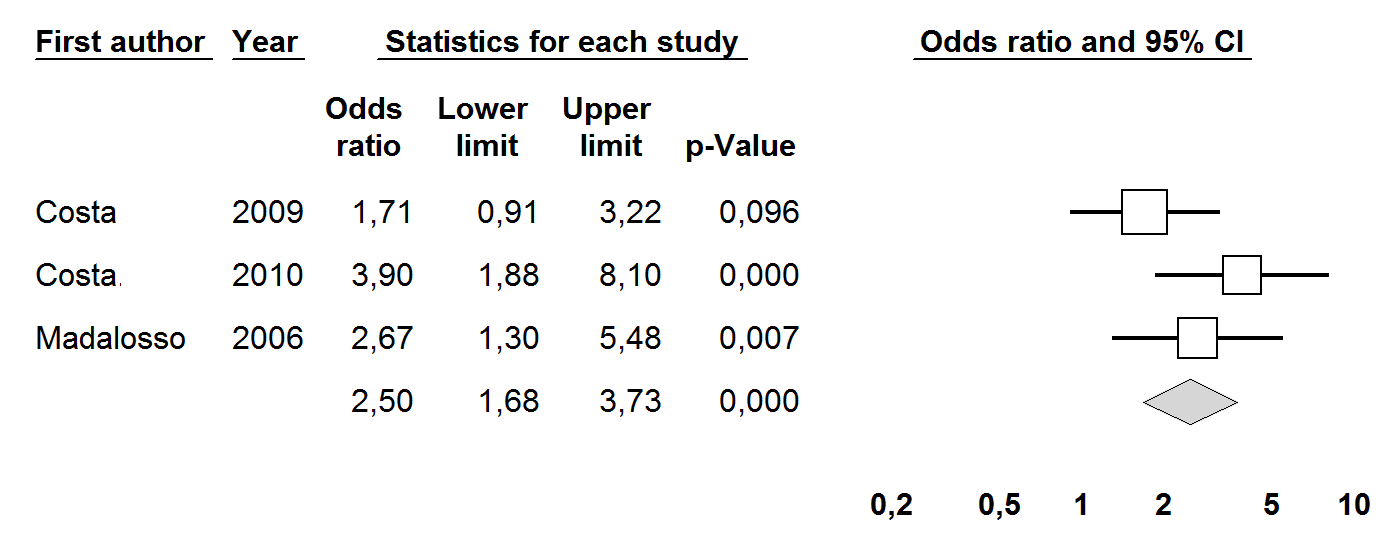


**XVI. Interval between the onset of fever and diagnosis (> 48 or > 60 days; results only for adults)**

**
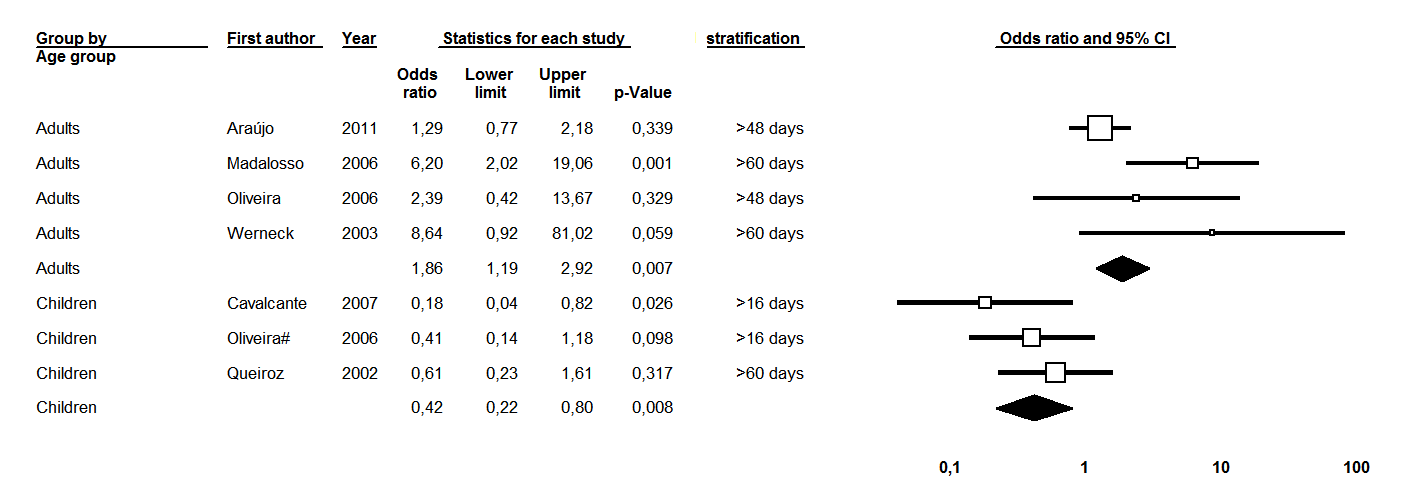
**

**XVII. Pulmonary rales**

**
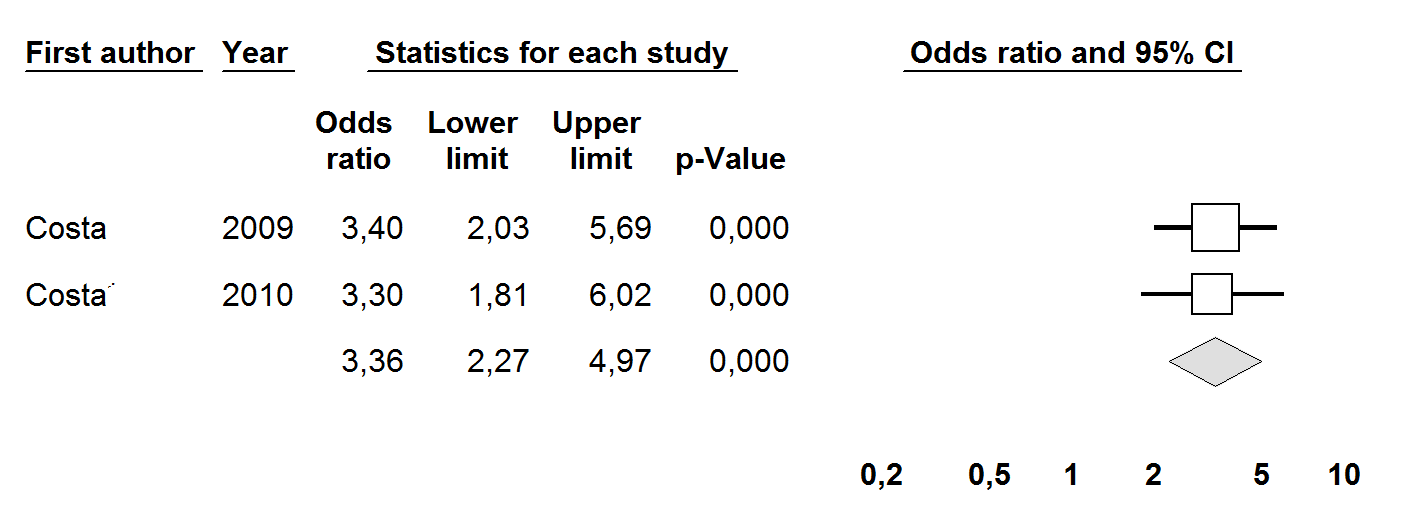
**

**XVIII. Serum albumin (< 3.0 g/dL)**

**
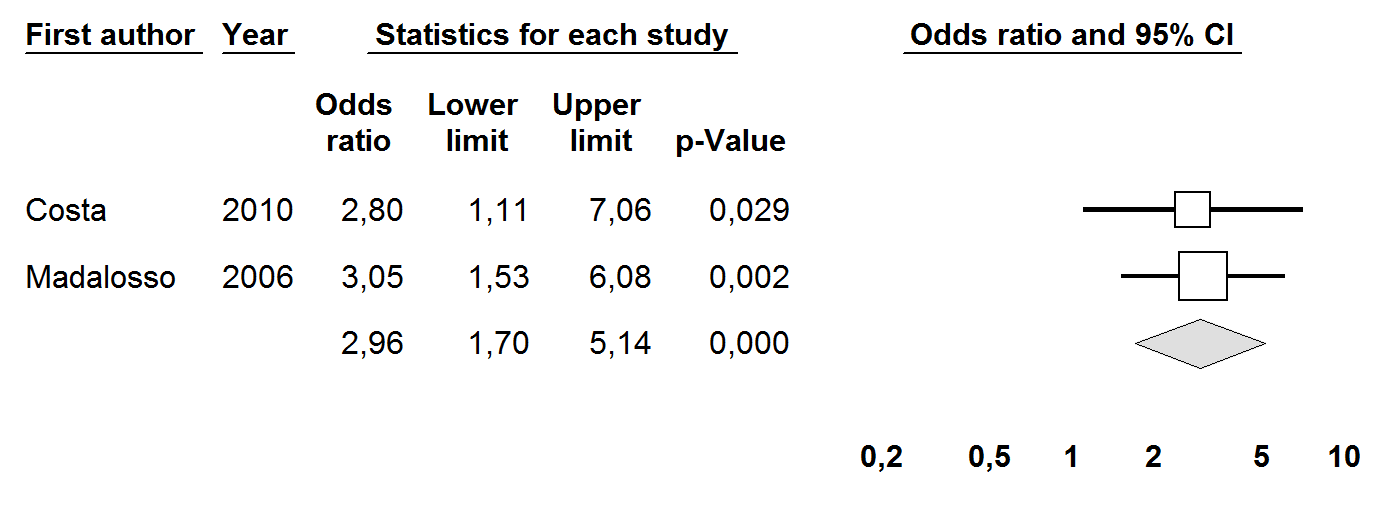
**

**XIX. Pallor**

**
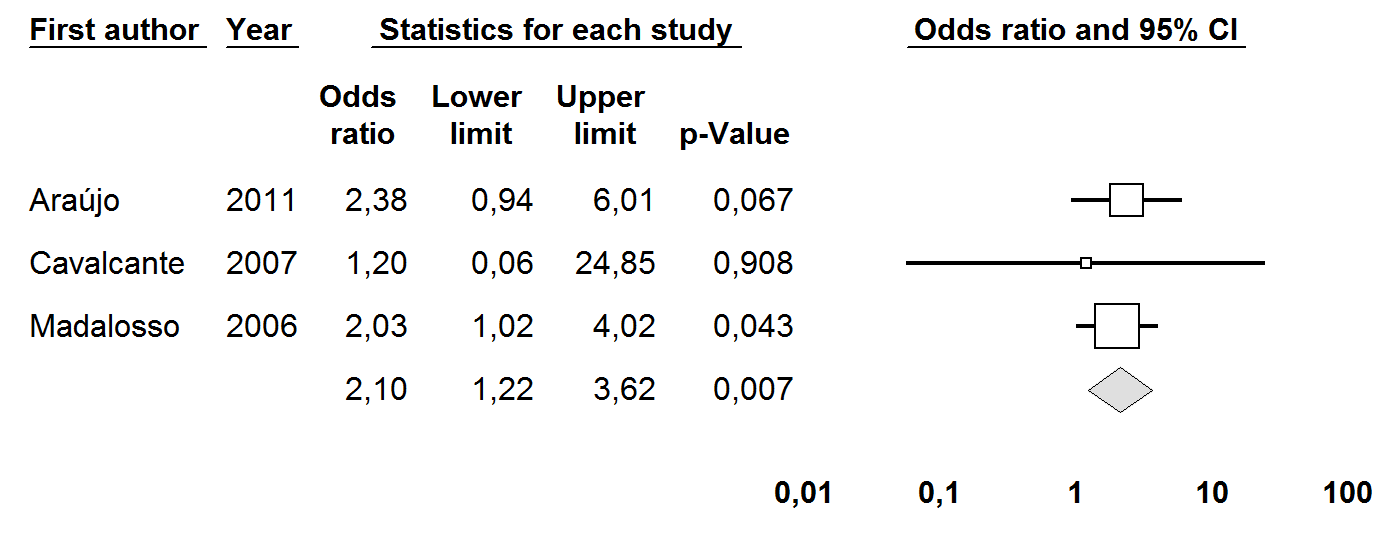
**

**XX. Cough**

**
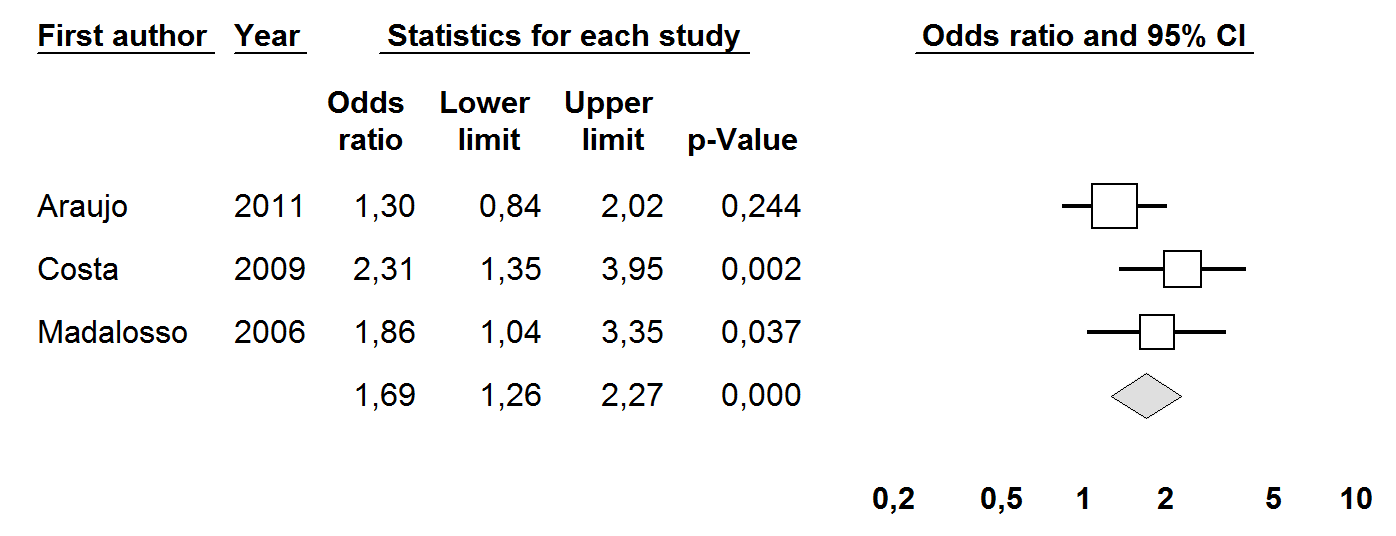
**

**XXI. Drowsiness**

**
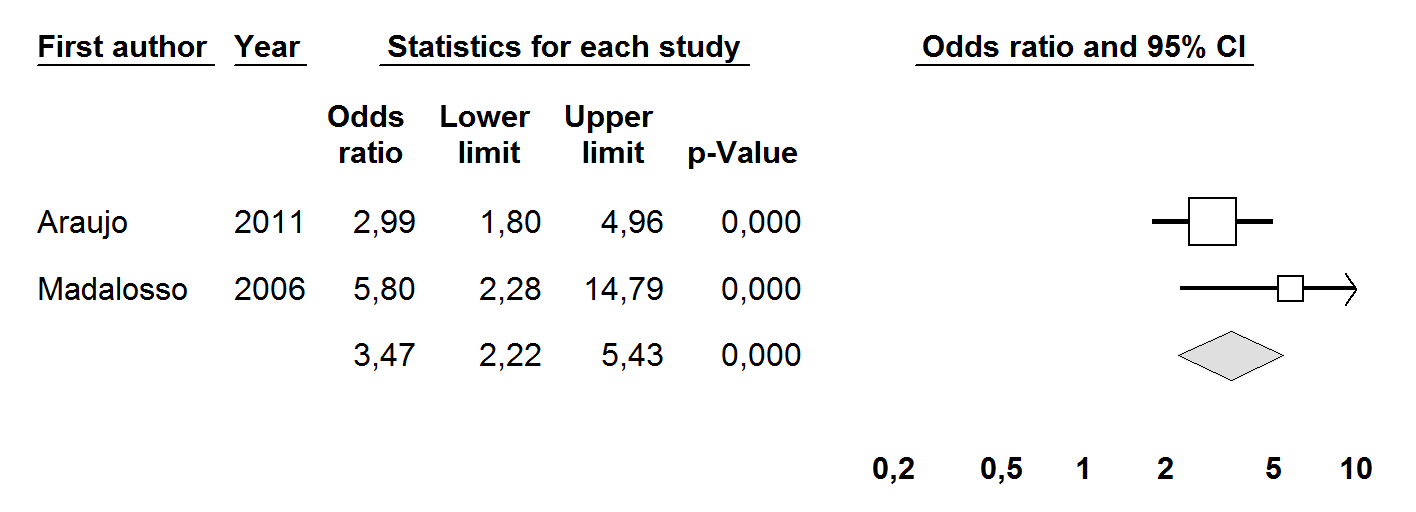
**

**XXII. Altered thorax X-ray**

**
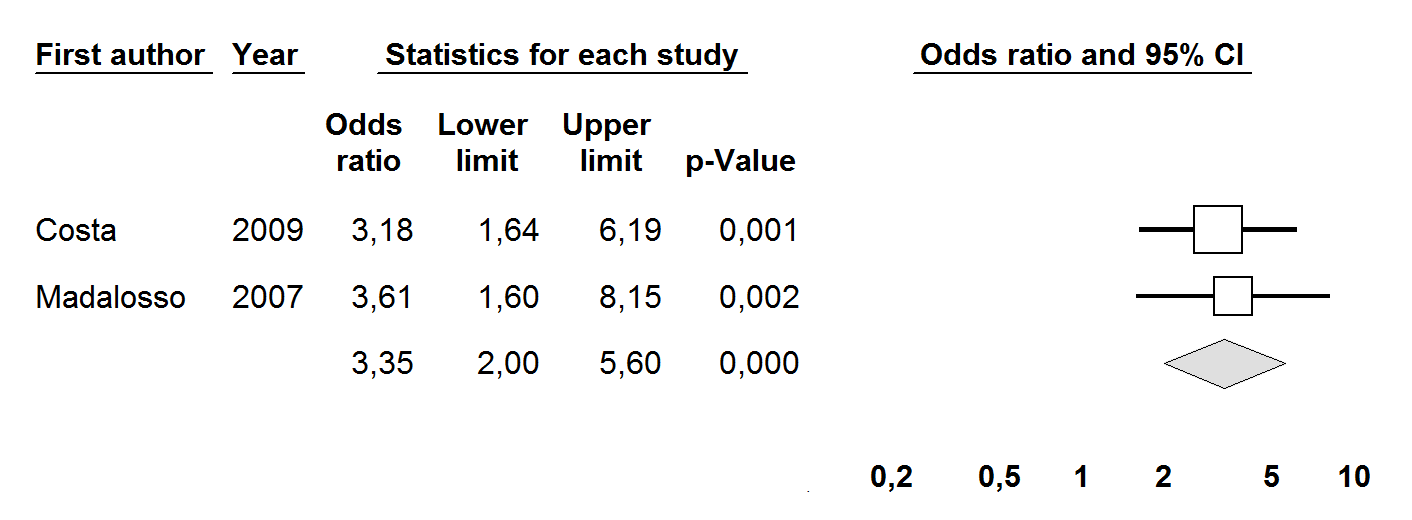
**

**XXIII. Gender ( referenced to females, OR = 1.0)**

**
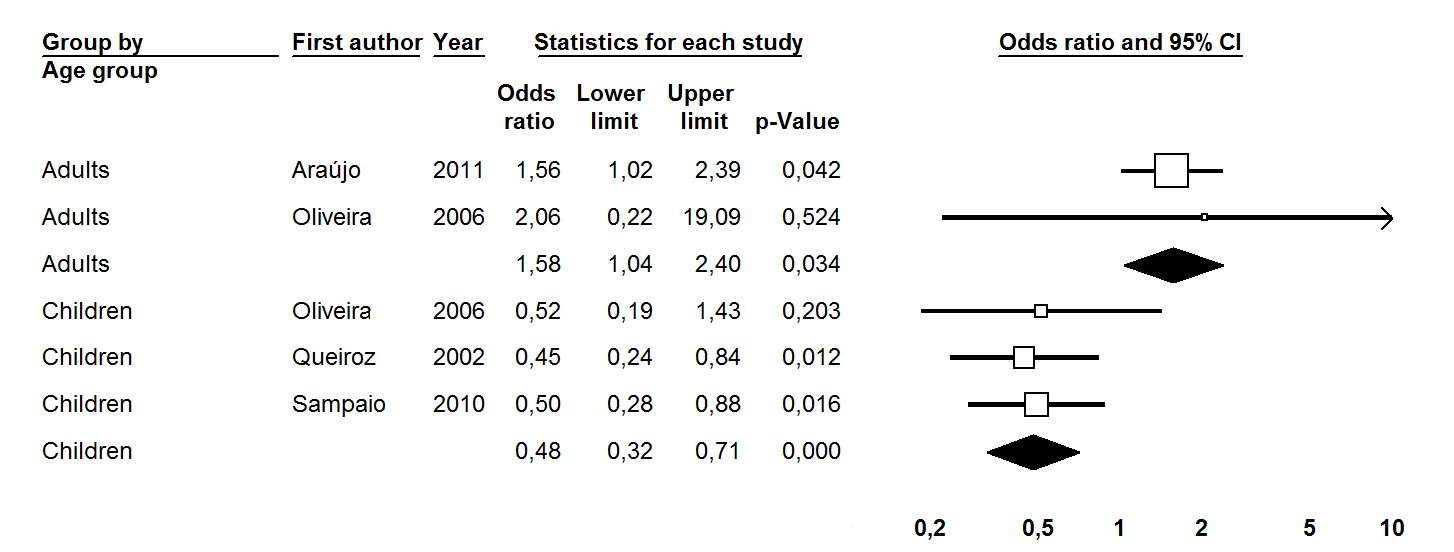
**

**XXIV. Fever**

**
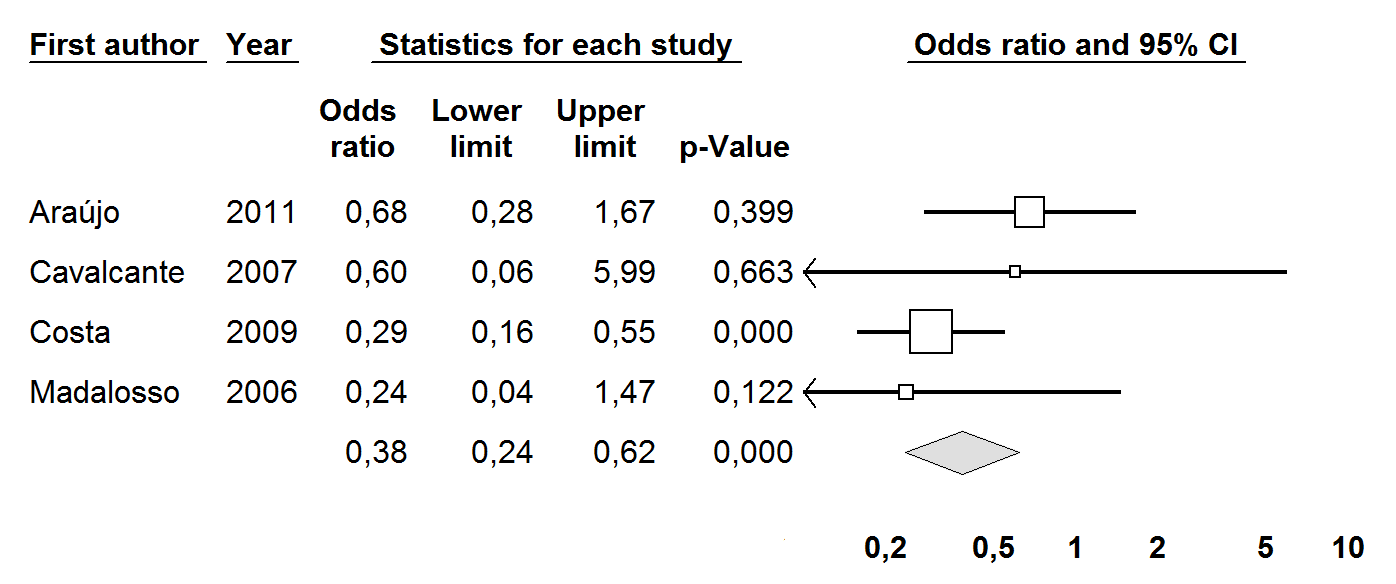
**

**XXV. Hepatomegaly (liver size > defined cut-off point)**

**
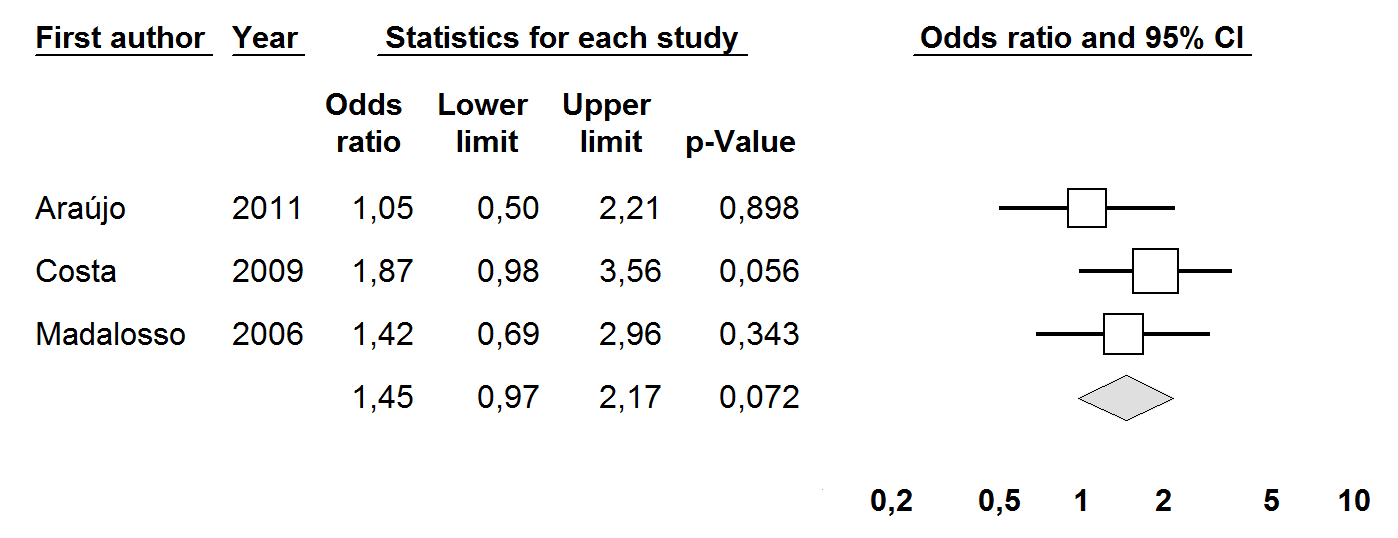
**

**XXVI. Weight loss**

**
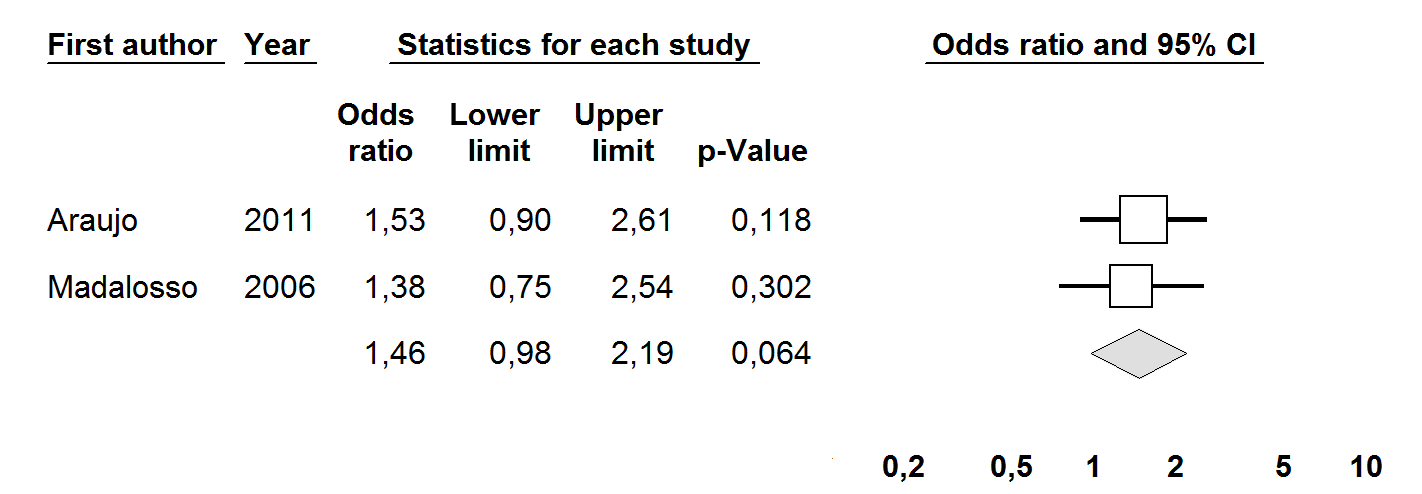
**

**XXVII. Splenomegaly (spleen size > defined cut-off point)**

**
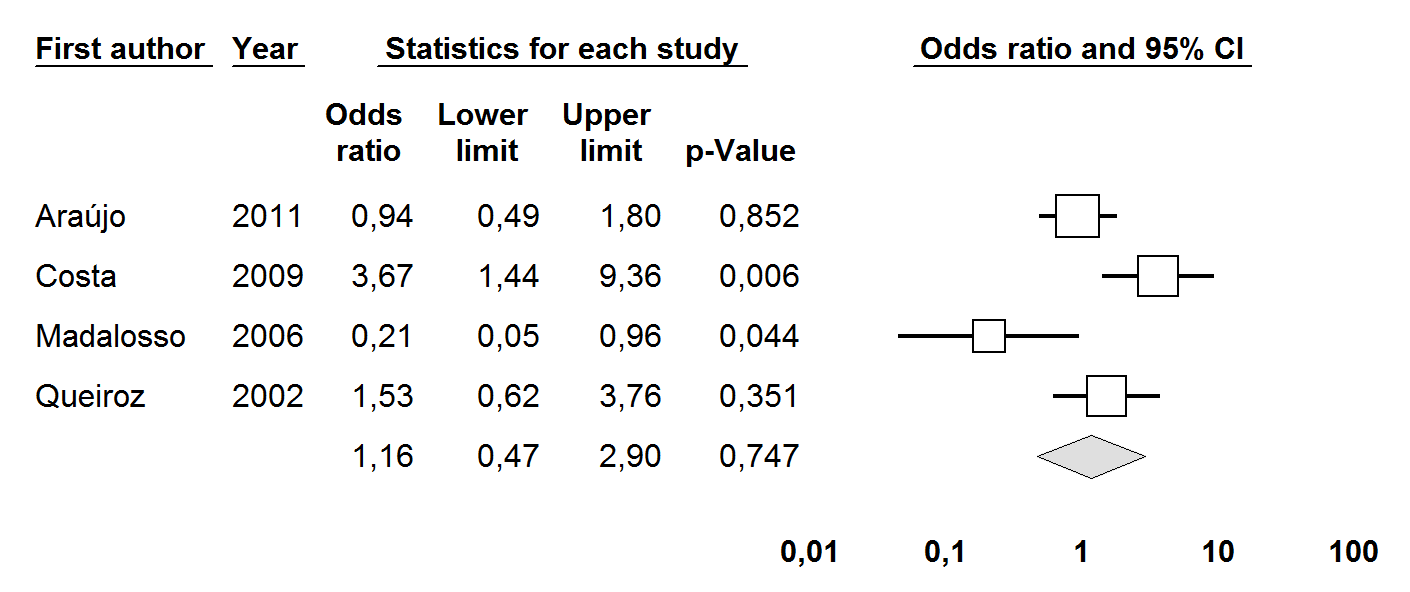
**
